# Supplementary material for: Infection prevention and control measures to reduce the transmission of mpox: A systematic review
Source: PLOS Glob Public Health. 2024 Jan 18;4(1):e0002731. doi: 10.1371/journal.pgph.0002731 (PMC10796032; doi:10.1371/journal.pgph.0002731)
Supplement: S1 Table — (DOCX) [file pgph.0002731.s003.docx]

Table S1: Review Question 1

| **Review question 1. Does the use of respirator versus a medical mask when interacting with a confirmed/suspect mpox patient during the infectious period reduce mpox infections?** | |
| --- | --- |
| **Population** | Health worker caring for a confirmed/suspect mpox patient during the infectious period in a household, congregate living, or healthcare setting​. |
| **Intervention** | Respirator (eg N95, FFP2) in addition to contact and droplet precautions. |
| **Comparator** | Medical mask in addition to contact and droplet precautions. |
| **Outcome** | Mpox infection. |
