# Supplementary material for: Infection prevention and control measures to reduce the transmission of mpox: A systematic review
Source: PLOS Glob Public Health. 2024 Jan 18;4(1):e0002731. doi: 10.1371/journal.pgph.0002731 (PMC10796032; doi:10.1371/journal.pgph.0002731)
Supplement: S2 Table — (DOCX) [file pgph.0002731.s004.docx]

Table S2: Review Question 2

| **Review question 2. Does the use of an airborne precaution room versus an adequately ventilated room in a healthcare facility for a mpox patient in the infectious period reduce mpox infection in health workers or patients?** | |
| --- | --- |
| **Population** | Health worker caring for, or a patient in proximity to, a confirmed/suspect mpox patient during the infectious period in a healthcare setting.​  Subgroups:  In-patient and out-patient setting. |
| **Intervention** | Airborne precaution room is defined as a room with high ventilation rate and controlled direction of airflow. This is achieved by either mechanical^a^ or natural^b^ ventilation. ​ |
| **Comparator** | Adequately ventilated single room is a room or area that has an adequate ventilation rate^c^ without controlled direction of airflow. ​ |
| **Outcome** | Mpox infection in health worker or patient. |
| Footnotes:  ^a^ Mechanical ventilation to meet criteria for an airborne precaution room: ​  Airflow - negative pressure is created to control the direction of airflow. The ventilation rate should be at least 12 ACH. ​  ^b^ Natural ventilation to meet criteria for an airborne precaution room: ​  Airflow: the airflow should be directed to areas free of transit or should permit the rapid dilution of contaminated air into the surrounding areas and the open air.  ​The average ventilation rate should be 160 liters/second per patient.  ^c^ Adequate ventilation in a single room may be achieved by mechanical, natural or hybrid ventilation.  Mechanical ventilation rate: ​two outdoor ACH and at least six total ACH.  Natural ventilation rate: ​60l/s/patient ​  Hybrid (mixed mode) ventilation is a combination of both mechanical and natural ventilation​. It relies on natural driving forces to provide the desired (design) flow rate. ​Mechanical ventilation can be used when the natural ventilation flow rate is too low. | |
