# Supplementary material for: Infection prevention and control measures to reduce the transmission of mpox: A systematic review
Source: PLOS Glob Public Health. 2024 Jan 18;4(1):e0002731. doi: 10.1371/journal.pgph.0002731 (PMC10796032; doi:10.1371/journal.pgph.0002731)
Supplement: S3 Table — (DOCX) [file pgph.0002731.s005.docx]

Table S3: Review Question 3

| **Review question 3. Does isolating a person with mpox until all lesions are fully healed versus not isolating reduce mpox infections?** | |
| --- | --- |
| **Population** | Person interacting with confirmed mpox (by RT-PCR) during the infectious periodin household and community settings. |
| **Intervention** | Mpox patient does not isolate^a^ provided they cover all non-healed lesions and wear a mask. |
| **Comparator** | Mpox patient isolates^a^until all lesions are fully healed.^b^ |
| **Outcome** | Mpox infection. |
| **Footnotes:**  ^a^Isolation: the separation of infected people with a contagious disease from people who are not infected.  ^b^Fully healed: lesions have crusted, scabs have fallen off and a fresh layer of skin has formed underneath. | |
