# Supplementary material for: Infection prevention and control measures to reduce the transmission of mpox: A systematic review
Source: PLOS Glob Public Health. 2024 Jan 18;4(1):e0002731. doi: 10.1371/journal.pgph.0002731 (PMC10796032; doi:10.1371/journal.pgph.0002731)
Supplement: S4 Table — (DOCX) [file pgph.0002731.s006.docx]

Table S4: Incident cases of mpox by clade with multiple or unknown routes of transmission resulting in infection

| Incident cases of mpox, by route of transmission and clade  Setting: All settings  Transmission: Multiple and unknown route of transmission | | | | | | |
| --- | --- | --- | --- | --- | --- | --- |
| Transmission route | Clade I  number/ total cases^b^ | Clade IIa  number/ total cases^c^ | Clade IIb  number/ total cases^d^ | Likely Clade IIb^a^  number/ total cases^e^ | Clade not reported in 2022  number/ total cases^f^ | Clade not reported before 2022  number/ total cases^g^ |
| Multiple routes^h^ | 18/20  (90.0%) | 8/79  (10.1%) | 1 /2  (50.0%) | 129/177  (72.9%) | 36/136  (26.5%) | 808/1517  (53.3%) |
| Unknown^i^ | 2/20  (10.0%) | 71/79  (89.9%) | 1 /2  (50.0%) | 48/177  27.1%) | 100/136  (73.5%) | 709/1517  (46.7%) |
| Total cases  (1931)^j^ | 20 | 79 | 2 | 177 | 136 | 1517 |
| Footnotes:  Incident cases are defined as an individual changing from a state of non-disease to disease over a specific period of time reported within an included study. ^a^Clade IIb is the primary variant largely circulating in the 2022 global mpox outbreak. These cases were reported in included studies in 2022 as West African clade before the change in clade nomenclature in August 2022. Since they occurred in 2022, it is assumed the cases are likely to be clade IIb.^b^Denominator calculated as the sum of all reported Clade I cases of mpox with multiple or unknown routes of transmission.^c^Denominator calculated as the sum of all reported Clade IIa cases of mpox with multiple or unknown routes of transmission.^d^Denominator calculated as the sum of all reported Clade IIb cases of mpox with multiple or unknown routes of transmission.^e^Denominator calculated as the sum of all cases of mpox reported West African clade in 2022 with multiple or unknown routes of transmission.^f^Denominator calculated as the sum of all mpox cases without a clade in 2022 with multiple or unknown routes of transmission.^g^Denominator calculated as the sum of all mpox cases without a clade reported before 2022.^h^More than one route of transmission was identified as possible by review authors. Possible routes: direct sexual contact, direct physical non-sexual contact fomite, inhalation.^i^Insufficient information was reported in studies to assign or hypothesise any route of transmission by review authors^j^Total 1931 incident cases of mpox, with multiple or unknown routes of transmission. | | | | | | |
