# Supplementary material for: Infection prevention and control measures to reduce the transmission of mpox: A systematic review
Source: PLOS Glob Public Health. 2024 Jan 18;4(1):e0002731. doi: 10.1371/journal.pgph.0002731 (PMC10796032; doi:10.1371/journal.pgph.0002731)
Supplement: S5 Table — (DOCX) [file pgph.0002731.s007.docx]

Table S5: Clinical samples of viral isolation attempts from adults or children with confirmed mpox infection

| **Population:** Adults or children with confirmed mpox infection  **Setting:** Italy, Spain | | | | | |
| --- | --- | --- | --- | --- | --- |
| Sample type | No. of samples (no. of studies) | Proportion of samples from which viral isolation successful (%) | Days on which sampling performed  (range in days from symptom onset) | Days on which viral isolation successful  (range indays from symptom onset) | References |
| Saliva | 33 [1] | 22 of 33 (66.7%) | 2 to 9 | 3 to 9 | 129 |
| Oropharyngeal swabs | 4 [1] | 1 of 4 (25.0%) | 9 to 12 | day 9 | 72 |
