# Supplementary material for: Infection prevention and control measures to reduce the transmission of mpox: A systematic review
Source: PLOS Glob Public Health. 2024 Jan 18;4(1):e0002731. doi: 10.1371/journal.pgph.0002731 (PMC10796032; doi:10.1371/journal.pgph.0002731)
Supplement: S6 Table — (DOCX) [file pgph.0002731.s008.docx]

Table S6: Air sampling in environments occupied by adults with confirmed mpox infection

| **Reference** | **Day of sampling** | **Participants** | **Clade** | **Proportion of air samples positive by PCR** | **Proportion of air samples from which virus isolated** |
| --- | --- | --- | --- | --- | --- |
| 129 | 2-9 post symptom onset | 44 | IIb | 27/ 42  (64.3%) | 0/ 27  (0.0%) |
| 128 | 6-30 post symptom onset | 7 | IIb | 5 /11  (45.5%) | 1/1^a^  (100.0%) |
| **Footnotes**  ^a^Sample collected on day 9 from reported symptom onset | | | | | |
