# Supplementary material for: Infection prevention and control measures to reduce the transmission of mpox: A systematic review
Source: PLOS Glob Public Health. 2024 Jan 18;4(1):e0002731. doi: 10.1371/journal.pgph.0002731 (PMC10796032; doi:10.1371/journal.pgph.0002731)
Supplement: S7 Table — (DOCX) [file pgph.0002731.s009.docx]

Table S7: Mpox lesion clinical samples in which viral isolation was attempted

| **Population:** Adults or children with confirmed mpox infection  **Setting:** Europe, DRC, Ivory Coast, Nigeria | | | | | |
| --- | --- | --- | --- | --- | --- |
| Sample type | No. of samples  (no. of studies) | Proportion of samples from which viral isolation successful (%) | Days on which sampling performed, range (days from symptom onset) | Days on which viral isolation successful, range (days from symptom onset) | References |
| Skin lesion swabs^a^ (day of sampling reported)^b^ | 10 [4] | 8 of 10 (80.0%) | 5 to 15 | 5 to 15 | 61,62,72,78 |
| Skin lesion swabs^a^ (day of sampling not reported) | 199 [4] | 93 of 199 (46.7%) | Not reported | Not applicable | 24,127,130,131 |
| **Footnotes**  **^a^** Skin lesion sampling includes vesicle, pustule, vesicle fluid, crusts or unspecified swab of lesion | | | | | |
