# Supplementary material for: Infection prevention and control measures to reduce the transmission of mpox: A systematic review
Source: PLOS Glob Public Health. 2024 Jan 18;4(1):e0002731. doi: 10.1371/journal.pgph.0002731 (PMC10796032; doi:10.1371/journal.pgph.0002731)
Supplement: S8 Table — (DOCX) [file pgph.0002731.s010.docx]

Table S8: Surface Sampling in environments occupied by adults with confirmed mpox infection

| **Reference** | **Setting** | **Number of**  **Participants** | **Clade** | **Day of sampling** | **Proportion positive by viral isolation**  **(%)** |
| --- | --- | --- | --- | --- | --- |
| 132 | Household | 1 | IIa | 3 days after patient left | 6 of 10  (60.0%) |
| 73 | Household | 1 | IIa | 15 days after patient left | 7 of 31  (22.5%) |
| 133 | Healthcare | 2 | IIb | Day 4 of occupation | 3 of 40  (7.5%) |
| 128 | Healthcare | 7 | IIb | Day 6-30 after symptom onset | 1 of 3  (33.3%) |
| 134 | Household | 2 | IIb | Day 20 of isolation (ongoing symptoms) | 0 of 21  (0.0%) |
