# Supplementary material for: Infection prevention and control measures to reduce the transmission of mpox: A systematic review
Source: PLOS Glob Public Health. 2024 Jan 18;4(1):e0002731. doi: 10.1371/journal.pgph.0002731 (PMC10796032; doi:10.1371/journal.pgph.0002731)
Supplement: S1 Appendix — (DOCX) [file pgph.0002731.s011.docx]

**Appendix 1: Search Strategy**

Search: Monkeypox virus only

**Ovid MEDLINE(R) and In-Process, In-Data-Review & Other Non-Indexed Citations**

**and Embase (OVID)**

Date of search: 8^th^ September 2022

1 Monkeypox virus/ or Monkeypox/

2 monkeypox.ti,ab.

3 monkey pox.mp.

4 1 or 2 or 3

**Biosis previews (Web of Science) and CAB Abstracts (Web of science):**

Date of search: 8^th^ September 2022

Topic = monkeypox*

**Global Index Medicus**

Date of search: 26^th^ September 2022

Search term: “monkeypox”

Search: Mpox-like viruses

**Ovid MEDLINE(R) and In-Process, In-Data-Review & Other Non-Indexed Citations <1946 to September 27, 2022>**

Date of search: 28^th^ September 2022

1 orthopoxvirus/ or cowpox virus/ or ectromelia virus/ or vaccinia virus/ or variola virus/

2 (orthopox* or cowpox or vaccinia or variola or buffalopox).tw.

3 1 or 2

8 Masks/ or mask*.mp.

9 Ventilation/ or ventilation.mp.

10 air quality.mp.

11 patient isolation.mp. or Patient Isolation/

12 8 or 9 or 10 or 11

14 3 and 12

15 nosocomial transmission.mp. or Infection Control/

16 hospital transmission.mp.

17 15 or 16

18 3 and 17

19 18 or 14

**Embase 1947-Present,**

Date of search: 28^th^ September 2022

1 orthopoxvirus/ or cowpox virus/ or ectromelia virus/ or vaccinia virus/ or variola virus/

2 (orthopox* or cowpox or vaccinia or variola or buffalopox).tw.

3 1 or 2

4 Masks/ or mask*.mp.

5 Ventilation/ or ventilation.mp.

6 air quality.mp.

7 patient isolation.mp. or Patient Isolation/

8 4 or 5 or 6 or 7

9 3 and 8

10 nosocomial transmission.mp. or Infection Control/

11 hospital transmission.mp.

12 10 or 11

13 3 and 12

15 9 or 13

16 monkeypox.m_titl.

17 15 not 16

**Web of Science (BIOSIS Previews, CABI: CAB Abstracts)**

Publication year range: 1900 to the present

Date of search: 28^th^ September 2022

#6 #4 OR #5

#5 #1 AND #3

#4 #1 AND #2

#3 "nosocomial transmission" or "Infection Control" or "hospital transmission" (Topic)

#2 mask* or Ventilation or "air quality" or "patient isolation" (Topic)

#1 orthopoxvirus* or cowpox or vaccinia or variola or buffalopox (Topic)

**Global Index medicus**

Publication year range: Earliest available to present

Date of search: 28^th^ September 2022

tw:((tw:(orthopoxvirus* OR cowpox OR vaccinia OR variola OR buffalopox)) AND (tw:(mask* OR ventilation OR "air quality" OR "patient isolation" OR "nosocomial transmission" OR "Infection Control" OR "hospital transmission" )))
