# Supplementary material for: Infection prevention and control measures to reduce the transmission of mpox: A systematic review
Source: PLOS Glob Public Health. 2024 Jan 18;4(1):e0002731. doi: 10.1371/journal.pgph.0002731 (PMC10796032; doi:10.1371/journal.pgph.0002731)
Supplement: S2 Appendix — (DOCX) [file pgph.0002731.s012.docx]

# Appendix 2: Quality Appraisal Questions

1. Are the case(s) so atypical that they would lead you to consider them as not representative?

2. Did the study adequately describe how cases were identified?

3. Did authors perform active case seeking?

4. Was the outcome (route of transmission) reported for all or nearly all cases?
