# Supplementary material for: Infection prevention and control measures to reduce the transmission of mpox: A systematic review
Source: PLOS Glob Public Health. 2024 Jan 18;4(1):e0002731. doi: 10.1371/journal.pgph.0002731 (PMC10796032; doi:10.1371/journal.pgph.0002731)
Supplement: S3 Appendix — (DOCX) [file pgph.0002731.s013.docx]

**Appendix 3: Quality Appraisal Results**

| **Reference** | **Are the case(s) so atypical that they would lead you to consider them as not representative?** | **Did the study adequately describe how cases were identified?** | **Did authors perform active case seeking?** | **Was the outcome (route of transmission) reported for all or nearly all cases?** |
| --- | --- | --- | --- | --- |
| 13 | No | No | No | No |
| 14 | No | Yes | Yes | No |
| 15 | No | Yes | Yes | Yes |
| 16 | No | Yes | No | Yes |
| 17 | No | Yes | Yes | Yes |
| 18 | No | Yes | Yes | Yes |
| 19 | No | Yes | Yes | Yes |
| 20 | No | Yes | Yes | Yes |
| 21 | No | Yes | No | Yes |
| 22 | No | No | No | Yes |
| 23 | No | Yes | No | No |
| 24 | No | Yes | No | Yes |
| 25 | No | Yes | No | Yes |
| 26 | No | Yes | No | No |
| 27 | No | Yes | No | Yes |
| 28 | No | Yes | Yes | Yes |
| 29 | No | Yes | No | No |
| 30 | No | Yes | Yes | No |
| 31 | No | Yes | No | No |
| 32 | No | Yes | No | Yes |
| 33 | No | Yes | No | Yes |
| 34 | No | Yes | Yes | Yes |
| 35 | No | Yes | Yes | No |
| 36 | No | Yes | No | Yes |
| 37 | Yes | Yes | No | Yes |
| 38 | No | Yes | No | Yes |
| 39 | No | Yes | No | Yes |
| 40 | No | Yes | No | No |
| 41 | No | Yes | No | Yes |
| 42 | No | Yes | Yes | Yes |
| 43 | No | Yes | No | Yes |
| 44 | No | Yes | No | No |
| 45 | No | No | Yes | Yes |
| 46 | No | Yes | Yes | No |
| 47 | No | Yes | Yes | Yes |
| 48 | No | No | No | No |
| 49 | No | Yes | No | Yes |
| 50 | No | Yes | Yes | Yes |
| 51 | No | Yes | Yes | Yes |
| 52 | No | Yes | Yes | Yes |
| 52 | No | Yes | No | Yes |
| 53 | No | Yes | Yes | Yes |
| 55 | No | Yes | Yes | Yes |
| 56 | No | Yes | Yes | No |
| 57 | No | Yes | No | Yes |
| 58 | No | Yes | No | Yes |
| 59 | No | Yes | No | Yes |
| 60 | No | Yes | Yes | Yes |
| 61 | No | Yes | Yes | Yes |
| 62 | No | Yes | No | Yes |
| 63 | No | Yes | No | Yes |
| 64 | No | Yes | No | Yes |
| 65 | No | Yes | Yes | Yes |
| 66 | No | Yes | Yes | Yes |
| 67 | No | Yes | Yes | No |
| 68 | No | Yes | No | Yes |
| 69 | No | Yes | Yes | No |
| 70 | No | Yes | Yes | No |
| 71 | No | Yes | Yes | Yes |
| 72 | No | Yes | Yes | No |
| 73 | No | Yes | No | Yes |
| 74 | No | Yes | No | Yes |
| 75 | No | Yes | No | Yes |
| 76 | No | Yes | No | Yes |
| 77 | No | Yes | Yes | No |
| 78 | No | Yes | No | Yes |
| 79 | No | Yes | No | Yes |
| 80 | No | Yes | Yes | No |
| 81 | Yes | Yes | Yes | Yes |
| 82 | No | Yes | No | Yes |
| 83 | No | Yes | No | Yes |
| 84 | No | Yes | No | Yes |
| 85 | No | Yes | No | Yes |
| 86 | No | Yes | Yes | Yes |
| 87 | No | Yes | Yes | Yes |
| 88 | No | Yes | Yes | Yes |
| 89 | No | Yes | No | Yes |
| 90 | No | Yes | No | No |
| 91 | No | Yes | No | Yes |
| 92 | No | No | Yes | Yes |
| 93 | No | Yes | No | Yes |
| 94 | No | No | No | Yes |
| 95 | No | Yes | Yes | No |
| 96 | No | Yes | Yes | Yes |
| 97 | No | Yes | Yes | Yes |
| 98 | No | Yes | No | Yes |
| 99 | No | Yes | Yes | No |
| 100 | No | No | No | Yes |
| 101 | No | Yes | Yes | No |
| 102 | No | Yes | No | Yes |
| 103 | No | Yes | Yes | Yes |
| 104 | No | No | No | Yes |
| 105 | No | Yes | No | Yes |
| 106 | No | Yes | No | Yes |
| 107 | No | Yes | No | Yes |
| 108 | No | Yes | Yes | Yes |
| 109 | No | Yes | No | Yes |
| 110 | No | Yes | No | Yes |
| 111 | No | No | No | Yes |
| 112 | No | Yes | No | Yes |
| 113 | No | Yes | No | Yes |
| 114 | No | No | Yes | Yes |
| 115 | No | Yes | Yes | No |
| 116 | No | Yes | No | Yes |
| 117 | No | Yes | Yes | Yes |
| 118 | No | Yes | Yes | Yes |
| 119 | No | Yes | No | No |
| 120 | No | Yes | Yes | Yes |
| 121 | No | Yes | Yes | No |
| 122 | No | Yes | Yes | Yes |
| 123 | No | Yes | Yes | No |
| 124 | No | Yes | No | Yes |
| 125 | No | Yes | No | Yes |
| 126 | No | Yes | No | Yes |
