# Supplementary material for: Infection prevention and control measures to reduce the transmission of mpox: A systematic review
Source: PLOS Glob Public Health. 2024 Jan 18;4(1):e0002731. doi: 10.1371/journal.pgph.0002731 (PMC10796032; doi:10.1371/journal.pgph.0002731)
Supplement: Attachment — Submitted filename: PGPH-D-23-00439_reviewer.pdf [file pgph.0002731.s014.pdf]

PLOS Global Public Health

Infection prevention and control measures to reduce the transmission of mpox: a systematic review

--Manuscript Draft--

**Manuscript Number:**	PGPH-D-23-00439
**Article Type:**	Research Article
**Full Title:**	Infection prevention and control measures to reduce the transmission of mpox: a systematic review
**Short Title:**	Infection prevention and control measures to reduce mpox transmission: a systematic review
**Corresponding Author:**	Rebecca Kuehn<br>Liverpool School of Tropical Medicine<br>Liverpool, Liverpool UNITED KINGDOM
**Order of Authors:**	Rebecca Kuehn
	Tilly Fox
	Gordon Guyatt
	Vittoria Lutje
	Susan Gould
**Keywords:**	MPOX; Mpox virus; Monkeypox; Infection prevention control,
**Abstract:**	Objectives<br><br>To assess the effectiveness of respiratory interventions and case isolation measures in reducing or preventing the transmission of mpox in humans and to inform future focused reviews on mpox transmission.<br><br>Methods<br><br>The WHO Clinical Management and Infection Prevention and Control guideline 2022 development group developed three structured research questions concerning respiratory and isolation infection prevention control measures for mpox. We conducted a systematic review that included a broad search of five electronic databases. In a two-stage process, we initially sought only randomized controlled trials and observational comparative studies; when the search failed to yield eligible studies, the subsequent search included all study designs including clinical and environmental sampling studies.<br><br>Results<br><br>To inform the questions the review team synthesized route of transmission data in mpox. There were 2420/3924 (61.7%) cases in which investigators identified transmission occurring through direct physical sexual contact. There proved to be no reported mpox cases in which investigators identified inhalation as a single route of transmission. There were 2/3924 (0.05%) cases in which investigators identified fomite as a single route of transmission. Clinical and environmental sampling studies generally failed to isolate mpox virus in saliva, oropharangeal swabs, mpox skin lesions, and hospital room air.<br><br>Conclusions<br><br>Current findings provide compelling evidence that transmission of mpox occurs through direct physical contact. Because investigators have not reported any cases of transmission via inhalation alone the impact of respiratory infection prevention control measures in reducing transmission will be minimal. Avoiding physical contact with others, covering mpox lesions and wearing a medical mask is likely to reduce onward mpox transmission; there may be minimal reduction in transmission from additionally

Powered by Editorial Manager® and ProduXion Manager® from Aries Systems Corporation

	physically isolating patients.
**Additional Information:**	
**Question**	**Response**
**Financial Disclosure**<br><br>Enter a financial disclosure statement that describes the sources of funding for the work included in this submission and the role the funder(s) played. This includes grants and any commercial funding of the work or authors.<br><br>This statement will be typeset if the manuscript is accepted for publication.<br><br>*Please review the submission guidelines and the instructions link below for detailed requirements and guidance.*	Rebecca Kuehn is supported by the Research, Evidence and Development Initiative (READ-It). READ-It (project number 300342-104) is funded by UK aid from the UK government; however, the views expressed do not necessarily reflect the UK government's official policies.<br><br>Tilly Fox is supported by the Research, Evidence and Development Initiative (READ-It). READ-It (project number 300342-104) is funded by UK aid from the UK government; however, the views expressed do not necessarily reflect the UK government's official policies.<br><br>Vittoria Lutje is supported by the Research, Evidence and Development Initiative (READ-It). READ-It (project number 300342-104) is funded by UK aid from the UK government; however, the views expressed do not necessarily reflect the UK government's official policies.<br><br>Gordon Guyatt received no specific funding for this work.<br><br>Susan Gould received no specific funding for this work.
**Competing Interests**<br><br>On behalf of all authors, disclose any competing interests that could be perceived to bias this work.<br><br>This statement will be typeset if the manuscript is accepted for publication.<br><br>*Please review the instructions link below and PLOS Global Public Health's competing interests policy to determine what information must be disclosed at submission.*	All authors declare no known competing interests
**Data Availability**<br><br>Before publication, Authors are required to make fully available and without restriction all data underlying their findings. Please see our PLOS Data Policy page for detailed information on this policy.	All data underlying the findings reported in the manuscript is provided as part of the submitted article.

A **Data Availability Statement**, detailing where the data can be accessed, is required at first submission. Insert your Data Availability Statement in the box below.<br><br>Please see the data reporting section of our submission guidelines for instructions on what you need to include in your Data Availability Statement.<br><br>This statement will be typeset if the manuscript is accepted for publication.<br><br>*PLOS allows rare exemptions to address legal and ethical concerns. If you have legal or ethical restrictions, please use the box below to detail these in full sentences for the Journal team to consider.*	

Manuscript Click here to access/download;Manuscript;Mpox13.03.2023.pdf

Title: Infection prevention and control measures to reduce the transmission of mpox: a systematic review

Author names and affiliations:

1. Rebecca Kuehn (corresponding author): Department of Clinical Sciences, Liverpool School of Tropical Medicine, Pembroke Place, Liverpool L3 5QA, United Kingdom

   Email: Rebecca.kuehn@lstmed.ac.uk

2. Tilly Fox: Department of Clinical Sciences, Liverpool School of Tropical Medicine, Pembroke Place, Liverpool L3 5QA, United Kingdom
3. Gordon Guyatt: Department of Health Research Methods, Evidence and Impact, McMaster University, Hamilton, Ontario, Canada
4. Vittoria Lutje: Department of Clinical Sciences, Liverpool School of Tropical Medicine, Pembroke Place, Liverpool L3 5QA, United Kingdom
5. Susan Gould: Department of Clinical Sciences, Liverpool School of Tropical Medicine, Pembroke Place, Liverpool L3 5QA, United Kingdom

Acknowledgments: Gemma Villaneuva, Anders Bach-Mortensen, Ferrucio Pelone.

Abstract

Objectives: To assess the effectiveness of respiratory interventions and case isolation measures in reducing or preventing the transmission of mpox in humans and to inform future focused reviews on mpox transmission.

Methods: The WHO Clinical Management and Infection Prevention and Control guideline 2022 development group developed three structured research questions concerning respiratory and isolation infection prevention control measures for mpox. We conducted a systematic review that included a broad search of five electronic databases. In a two-stage process, we initially sought only randomized controlled trials and observational comparative studies; when the search failed to yield eligible studies, the subsequent search included all study designs including clinical and environmental sampling studies.

Results: To inform the questions the review team synthesized route of transmission data in mpox. There were 2420/3924 (61.7%) cases in which investigators identified transmission occurring through direct physical sexual contact. There proved to be no reported mpox cases in which investigators identified inhalation as a single route of transmission. There were 2/3924 (0.05%) cases in which investigators identified fomite as a single route of transmission. Clinical and environmental sampling studies generally failed to isolate mpox virus in saliva, oropharangeal swabs, mpox skin lesions, and hospital room air.

Conclusions: Current findings provide compelling evidence that transmission of mpox occurs through direct phys[illegible]ontact. Because investigators have not reported any cases of transmission via inhalation alone the impact of respiratory infection prevention control measures in reducing transmission will be minimal. Avoiding physical contact with others, covering mpox lesions and wearing a medical mask is likely to reduce onward mpox transmission; there may be minimal reduction in transmission from additionally physically isolating patients.

Keywords

Mpox

Mpox virus

Monkeypox

Units and Abbreviations

ACH: Air changes per hour

IPC: Infection Prevention Control

RT-PCR: Reverse transcriptase polymerase chain reaction

WHO: World Health Organization

Introduction

Mpox is a zoonotic disease caused by mpox virus, an enveloped double-stranded DNA virus in the *Orthopoxvirus* genus of the *Poxviridae* family. The World Health Organization (WHO) declared mpox (then termed monkeypox) a Public Health Emergency of International Concern (PHEIC) on July 23rd, 2022. The 2022 mpox outbreak was associated with sustained human-to-human transmission that had not been previously described; historically mpox occurred primarily in west and central Africa, with infection commonly reported in persons who had contact with probable animal reservoirs with cases of secondary transmission reported in household contacts. Incidence in endemic areas of central and West Africa had been increasing rapidly over the past decades, coinciding with cessation of smallpox vaccination and eradication programmes.

Mpox incubates between five to 21 days and typically presents symptoms in two stages: the invasion period lasting from zero to five days characterized by fever, headache, lymphadenopathy, back pain, myalgia, and asthenia; following this, skin symptoms may appear between one to three days from onset of fever, with a rash evolving from macules to papules, vesicles, pustules and then crusts, often affecting the face, extremities, oral mucous membranes and genitalia.

The effectiveness of any infection prevention and control measures for mpox will depend on route(s) of transmission. Suspected or confirmed transmission routes include direct physical contact with an infected patient (non-sexual physical contact or sexual physical contact), indirect contact (fomite transmission) inhalation of fomites or infectious droplets and transplacental transmission. Human infection is also possible from contact with infected animals (scratches, bites, preparing, eating or using infected meat and animal products). Areas of uncertainty exist concerning the potential for asymptomatic transmission or the transmission potential of other possible routes, such as breastmilk, semen, vaginal fluids, urine, faeces or insect vectors.

There is a need for effective interventions to prevent the transmission of mpox. The WHO Clinical Management and Infection Prevention and Control 2022 guideline development group developed two research questions concerning airborne and respiratory infection prevention and control interventions and one question concerning case physical isolation interventions in mpox. It was expected that scarce evidence, if any, from randomized controlled trials or comparative interventional trials to inform the research questions would exist. As such, it was anticipated that the review questions would be informed indirectly using data on the number of incident cases of mpox by route of transmission and clinical and environmental sampling studies demonstrating viral culture positivity. This is based on the inference that, if there are a significant number of cases transmitted by inhalation or fomites and/or strong viral culture positivity from samples representative of these routes of transmission, interventions directed at preventing transmission via these route(s) would be of importance. Therefore, we conducted a systematic review to provide evidence for guideline recommendations regarding use of respiratory barriers to mpox transmission and physical isolation of mpox patients.

Objective

To assess the effectiveness of respiratory and case isolation infection precaution measures in reducing or preventing the transmission of mpox in humans.

Methods

1. Interventions for preventing transmission of mpox virus.

The review research questions developed by WHO Clinical Management and Infection Prevention and Control 2022 guideline development group for this review were:

**Review question 1. Does the use of respirator versus a medical mask when interacting with a confirmed/suspect mpox patient during the infectious period reduce mpox infections?**	
**Population**	Health worker caring for a confirmed/suspect mpox patient during the infectious period in a household, congregate living or healthcare setting.
**Intervention**	Respirator (eg N95, FFP2) in addition to contact and droplet precautions
**Comparator**	Medical mask in addition to contact and droplet precautions
**Outcome**	Mpox infection

**Review question 2. Does the use of an airborne precaution room versus an adequately ventilated room in a healthcare facility for a mpox patient in the infectious period reduce mpox infection in health workers or patients?**	
**Population**	Health worker caring for, or patient in proximity to, a confirmed/suspect mpox patient during the infectious period in a healthcare setting.<br>Subpopulation-disaggregated by in-patient versus out-patient setting
**Intervention**	Airborne precaution room is defined as a room with high ventilation rate and controlled direction of airflow. This is achieved by either mechanical* or natural** ventilation.<br>*Mechanical ventilation:<br>• Airflow: negative pressure is created to control the direction of airflow.<br>• Ventilation rate: the ventilation rate should be at least 12 ACH.<br>**Natural ventilation:<br>• Airflow: the airflow should be directed to areas free of transit, or should permit the rapid dilution of contaminated air into the surrounding areas and the open air;<br>Ventilation rate: the average ventilation rate should be 160 liters/second per patient
**Comparator**	Adequate[illegible]ntilated single room is a room or area that has an adequate ventilatio[illegible]e without controlled direction of airflow.<br>Mechanical ventilation:<br>• ventilation rate: two outdoor ACH and at least six total ACH<br>Natural ventilation:<br>• ventilation rate: 60l/s/patient<br>Hybrid (mixed mode) ventilation:<br>• combination of both mechanical and natural ventilation<br>• relies on natural driving forces to provide the desired (design) flow rate<br>• uses mechanical ventilation when the natural ventilation flow rate is too low
**Outcome**	Mpox infection in health worker or patient

Review question 3. Does isolating a person with mpox until all lesions are fully healed versus not isolating reduce mpox infections?	
**Population**	Person interacting with confirmed mpox (by RT-PCR) during the infectious period, household, and community settings.
**Intervention**	When all non-healed lesions are covered and patient wears a mask, mpox patient does not isolate* (physically separate from others)
**Comparator**	Mpox patient isolates* (physically separate from others) until all lesions are fully healed**
**Outcome**	Mpox infection
**Additional information**	***Isolation:** the separation of infected people with a contagious disease from people who are not infected.<br><br>***Fully healed:** lesions have crusted, scabs have fallen off and a fresh layer of skin has formed underneath

To provide direct evidence on these review questions, in collaboration with members of the WHO Clinical Management and Infection Prevention and Control 2022 guideline development, the author team developed a protocol and then undertook this systematic review process using the following methods:

Inclusion Criteria

Population

Humans with laboratory confirmed mpox infection; or
humans with laboratory confirmed mpox-like infection; or
humans with exposure to a laboratory confirmed mpox infection or mpox-like infection.

Mpox-like infection is defined for the purposes of the review as infection due to orthopox viruses other than mpox that are capable of human-to-human transmission, namely buffalopox, cowpox, vaccinia and variola.

Types of interventions

1. Respiratory precautions including medical masks or use of respirators.
2. Personal contact precautions including use of gloves, gowns, eye protection.
3. Isolation of cases
4. Ventilation including natural, mechanical, negative pressure gradient, positive pressure ventilated lobby.

The administration of any type of vaccine to health care workers or contacts of mpox or mpox-like virus confirmed patients was not to be considered as an intervention type.

Control

- No intervention or;
- Any different intervention measure used as a comparator to the intervention group in the study.

Outcomes

1. Confirmed secondary mpox or mpox-like virus infection expressed as an absolute number or rate of secondary transmission.
2. All reported adverse effects related to the interventions.

Settings
All countries and the following contexts were eligible for this review: households, congregate-living, community and healthcare settings.

Types of studies

- RCTs, controlled before-and-after studies, observational comparative studies in participants with confirmed mpox or exposed to mpox virus.
- RCTs or observational comparative studies in participants with exposure to or confirmed mpox-like virus infection.

Exclusion Criteria

1. Studies published in a language other than English.
2. Studies of designs other than RCTs, controlled before-and-after studies, or observational comparative studies for participants with confirmed mpox infection or exposure to mpox.
3. Studies of designs other than RCTs or observational comparative studies in participants with viruses other than viruses defined as mpox-like viruses.
4. Studies that do not include a review question-specific intervention to reduce or prevent the transmission of mpox or mpox-like viruses.
5. Studies conducted in animals.

Literature Search Strategy

Using broad search terms including terms for mpox-like viruses and without date or language limits, the search in September 2022 included the following databases: Medline (OVID), Embase (OVID), Biosis previews (Web of Science), CAB Abstracts (Web of science), and Global Index Medicus (Appendix 1). The author team instituted a call to topic experts for papers concerning the review questions for relevant studies up to 15th December 2022.

Selection of Studies

The team uploaded the results of the literature searches into Distiller SR (DistillerSR 2021) and multiple authors independently undertook screening of title and abstract literature search results according to the Cochrane Collaboration's Rapid Review Methods (Garritty 2020). [illegible]uthor assessed a study as eligible for full text screening; two authors assessed a study as requiring exclusion. Multiple authors independently undertook full text screening according to review inclusion criteria. One author assessed a study as eligible for inclusion for data extraction; two reviewers assessed a study for exclusion. Authors resolved disagreement at any stage by discussion.

2. Transmission of mpox virus

A second review protocol had been prospectively written and confirmed with members of the WHO Clinical Management and Infection Prevention and Control guideline development group in the event of no randomized controlled trials or comparative observational studies being identified in the first review (see methods section 1 'Interventions for preventing transmission of mpox virus' above).

This second review aimed to identify and synthesize evidence on transmission that could indirectly inform the research questions by answering the following:

1. What is the proportion of new incident cases of mpox disaggregated by route of transmission?
2. What is the infectious period of mpox, disaggregated by route of transmission?

The infectious period is defined as the number of days since the onset of symptoms.

Inclusion criteria

Population: Human participant of any age with laboratory confirmed mpox infection or exposure of a human to a laboratory-confirmed mpox patient or exposure to a suspected human mpox case.

The WHO definition of a suspected case of mpox infection was used (WHO 2022).

Laboratory confirmed infection was defined as reverse-transcriptase polymerase chain reaction (RT-PCR) positive or viral culture positive.

Outcomes:

1. Mpox infection

Type of study: any scientific article of any design including clinical and environmental sampling studies.

Setting: All countries and all contexts.

Exclusion criteria

1. Studies not including a human case of laboratory confirmed mpox infection or exposure to a laboratory-confirmed mpox patient or exposure to a suspected mpox case.
2. Studies concerning animal-to-animal mpox transmission or animal-to-human transmission.
3. Studies not published in English.
4. Experimental laboratory transmission studies.
5. Studies meeting the inclusion criteria but not otherwise containing information relevant to the review questions.

Literature Search strategy

The review team used the same search strategy as for methods section 1 ‘Interventions for reducing the risk of transmission of mpox virus’ (above).

Selection of studies

The results of the literature searches were uploaded into Distiller SR (DistillerSR 2021). Screening of results was undertaken according to the Cochrane Collaboration’s Rapid Review Methods (Garritty 2020). Title and abstract screening of all studies identified in the literature searches was undertaken independently by multiple authors; one author was required to assess a study as eligible for full text screening; two authors were required to assess a study as requiring exclusion. Full text screening against the review inclusion criteria was undertaken independently by multiple authors. One author was required to assess a study as eligible for inclusion to data extraction; two reviewers were required to assess a study for exclusion. Authors resolved disagreement at any stage by discussion.

Data extraction and management

Two authors extracted data from all included studies using a pre-piloted data extraction form within Distiller SR. One author extracted all relevant data and the second author cross-checked all extracted data. Data was extracted concerning characteristics of the study participants including number of primary and/or secondary cases, country, year of study, setting of transmission (such as household, healthcare), clade of mpox, reported nature of contact of participants to a potential or

confirmed course of mpox, study author reported modes or potential modes of transmission, and data concerning clinical or environmental sampling including sample type, and day of sample PCR or viral culture positivity from symptom onset.

Risk of bias assessment

No risk of bias assessment was undertaken; no randomized controlled trials or observational comparative studies had been identified.

Data synthesis

1. What is the proportion of new incident cases of mpox disaggregated by route of transmission?

Two authors independently categorized reported human mpox cases from identified studies by route(s) of transmission. Authors resolved disagreement at any stage by discussion. Authors assigned the following route(s) of transmission to each case as applicable: direct sexual physical contact, direct non-sexual physical contact, fomite, inhalation, transplacental, needlestick, ingestion or unknown.

The routes of transmission are defined as:

- Direct sexual physical contact: transmission occurring in the context of any type of sexual activity, including oral sex, penetrative anal or vaginal sex (insertive and receptive), or hand-to-genital contact.
- Direct non-sexual physical contact: direct physical touch with the exclusion of any sexual physical as defined above.
- Fomite: indirect contact transmission involving contact of a susceptible host with a contaminated object or surface.
- Inhalation: occurs when infectious particles, of any size, travel through the air, enter and are deposited at any point within the respiratory tract of a (susceptible) person. This form of transmission can occur when the infectious particles have travelled either a short- or long-range from the infected person.
- Transplacental: transmission via the placenta from mother to foetus.
- Percutaneous injury: transmission via percutaneous injury with a contaminated object, such as a needle.

The review team then categorised all cases into one of three categories: (i) single route of transmission reasonably identified, (ii) multiple routes of transmission possible, and (iii) unknown. The category of a single route of transmission applied when sufficient data concerning the case history, epidemiology and/or clinical details was reported to reasonably judge that a single route of transmission had occurred. The category of multiple possible routes of transmission applied when more than one route of transmission was judged as reasonably possible based on reported information. Authors applied the category of unknown when there was insufficient information reported in the study to assign or hypothesize any route of transmission.

The number of cases for each route and category of transmission is reported as a whole number and percentage of the total. Data are presented for each route of transmission category, by mpox clade, and by route of transmission in the healthcare and household settings. Data that could inform the subgroups within the research questions is summarized.

2. What is the infectious period of mpox, disaggregated by route of transmission?

Authors separated data from included studies into either human mpox clinical samples or environmental samples from an environment occupied by an mpox case. The review team assigned data concerning environmental air sampling, mask sampling, and upper respiratory tract clinical sampling to the review questions concerning prevention of airborne transmission. Data concerning environmental surface sampling and clinical sampling of active skin lesions were assigned to the review question concerning case isolation measures.

Within these categories the review team identified and summarized longitudinal studies and summarized cross-sectional studies that attempted viral isolation. The number of samples for each category is presented. Data that could inform the subgroups within the review questions is summarized.

Summary of findings and assessment of certainty of the evidence

A summary of findings table is presented for each review question. Data to inform the outcome of mpox infection is inferred from the number of reported mpox cases by route of transmission. Data from clinical and environmental sampling studies, representing lower quality evidence, is narratively summarized in the results section.

The rating of the certainty of evidence is based on the GRADE approach following the GRADE guidance as recommended in the *Cochrane Handbook for Systematic Reviews of Interventions* (Schünemann 2022).

Results

Search Results

The searches identified 2514 unique records. Authors assessed the full text of 725 studies; 127 studies were included and 598 studies were excluded. The study selection process is seen in Appendix 2.

Included studies

No randomized controlled trials or observational comparative studies were identified that directly informed any of the IPC review questions. No studies concerning mpox-like viruses met the inclusion criteria.

119 studies reported cases of human-to-human mpox transmission. 35 studies were published prior to the 2022 outbreak, and 84 studies were published during 2022. Of these, 46 studies concerned cases acquired from Africa, 42 studies from Europe, 16 studies from the USA, 5 studies from the United Kingdom, 3 studies from Brazil, 1 study from Canada and 6 studies did not report the country of acquisition of infection.

Included cases

There were 3924 cases of human-to-human transmission. Typical cases were males over the age of 18 years; it was not possible to disaggregate age and gender for 35% of cases (Table 1).

Table 1: Characteristics of reported cases

**Age**	**Male** Number of cases / total cases	**Female** Number of cases / total cases	**Non-binary** Number of cases / total cases	**Unable to be disaggregated** Number of cases / total cases
Under 18 years	15/3924 (0.4%)	10 /3924 (0.3%)	0 / 3924 (0%)	1372 / 3924 (35%)
18 years or older	2491/3924 (63.5%)	35 /3924 (0.9%)	1 /3924 (0.03%)	

Results: Review Question 1 and 2

Review questions 1 and 2 are considered together as they both concern respiratory infection prevention control interventions.

Summary of Findings Table 1: Does the use of respirator versus a medical mask reduce mpox transmission?

Population: Adults and children with mpox
Intervention: Respirator in addition to contact and droplet precautions
Comparator: Medical mask as part of contact and droplet precautions
Setting: Inpatient and outpatient

**Outcome**	**Study results and measurements**	**Absolute effect estimates transmission**		**Certainty of evidence**	**Plain language summary**
		**Medical mask** as part of contact and droplet precautions	**Respirators** in addition to contact and droplet precautions		
**Mpox infection** inferred from transmission route frequency data[a]	No reported cases of transmission by inhalation in 3924 patients (119 studies) Inferred odds ratio[b]: 1	Uncertain[c]	Uncertain, but no different to medical mask[d]	Moderate Due to serious indirectness	A respirator probably has no difference in preventing mpox transmission compared to a medical mask.

Footnotes
[a]No studies identified that directly informed the research question.
[b] As no cases of transmission through inhalation were identified it was inferred there would be no difference between the intervention and comparator groups.
[c]The baseline transmission risk was unable to be estimated due to complete lack of data.
[d]The risk in the intervention group was unable to be estimated due to an unknown baseline risk in the comparator group.

Summary of Findings Table 2: Does the use of an airborne precaution room versus an adequately ventilated room in a healthcare facility for a mpox patient reduce mpox infection in health workers or patients?

**Population:** Adults and children with mpox<br>**Intervention:** Airborne precaution room<br>**Comparator**: Adequately ventilated room<br>**Setting:** Inpatient and outpatient					
**Outcome**	**Study results and measurements**	**Absolute effect estimates transmission**		**Certainty of evidence**	**Plain language summary**
		**Adequately ventilated single room**	**Airborne precaution room**		
Mpox infection inferred from transmission route frequency data[a]	No reported cases of transmission by inhalation in 3924 patients (119 studies)<br><br>Inferred odds ratio[b]: 1	Uncertain[c]	Uncertain, but no different to adequately ventilated room[d]	Moderate<br><br>Due to serious indirectness	The use of an airborne precaution room probably has no impact on preventing mpox transmission compared to an adequately ventilated room

Footnotes

[a]No studies identified that directly informed the research question.

[b] As no cases of transmission through inhalation were identified it was inferred there would be no difference between the intervention and comparator groups.

[c]The baseline transmission risk was unable to be estimated due to complete lack of data.

[d]The risk in the intervention group was unable to be estimated due to an unknown baseline risk in the comparator group.

Number of incident cases by route of transmission

Where they could identify a single route of human-to-human transmission, investigators reported no cases in which inhalation could have been the exclusive mode of mpox virus transmission (Table 2).

Table 2: Incident cases of mpox by route of transmission

Population: Adults and children with confirmed mpox
Setting: All settings
Transmission: A single route of transmission was identified

Route of transmission	Number of cases/total cases	Number of studies
Direct sexual contact	2420/3924 (61.7%)	57
Physical touch (non-sexual)	35/3924 (0.9%)	2
Fomite	2/3924 (0.05%)	1
Transplacental	1/3924 (0.03%)	1
Percutaneous injury with contaminated object	3/3924 (0.1%)	3
Inhalation	0/3924 (0%)	0
Multiple routes [a]	929/3924 (23.7%)	28
Unknown [b]	552/3924 (14.0%)	44

Footnotes
[a] More than one route of transmission was identified as possible by authors. Possible transmission routes: direct sexual contact, direct physical non-sexual contact, fomite, inhalation.

[b] Insufficient information was reported in studies to assign or hypothesise any route of transmission.

Table 3 displays the clade of mpox by route of transmission in cases where a single route of transmission could be identified. Studies not reporting on clade have been split into those published in 2022 and those published prior to 2022. Cases reported in 2022 were inferred to be clade IIb since identification and naming of clade IIb did not occur until August 2022.

Table 3: Proportion of new incident cases of mpox, by route of transmission for each clade of mpox.

Population: Adults and children with confirmed mpox
Setting: All settings
Transmission: A single route of transmission was identified

	Clade I<br><br>number/ total cases	Clade IIa<br>(reported as WA pre-2022)<br><br>number/ total cases	Clade IIa<br>(reported as WA but occurring in 2022, probable IIb)<br><br>number/ total cases	Reported as Clade IIb<br><br>number/ total cases	Not reported Studies from 2022<br><br>number/total cases	Not reported, studies dating before 2022<br><br>number/total cases
Direct sexual contact	2/6	16/91	1079/1166	1/47	1322/1414	0/1200
Physical touch (non-sexual)	0/6	0/91	31/1166	0/47	4/1414	0/1200
Fomite	0/6	0/91	0/1166	2/47	0/1414	0/1200
Transplacental	0/6	0/91	0/1166	0/47	0/1414	1/1200
Inhalation	0/6	0/91	0/1166	0/47	0/1414	0/1200
Percutaneous injury	0/6	0/91	0/1166	0/47	3/1414	0/1200
Multiple routes[a]	1/6	0/91	8/1166	0/47	4/1414	916/1200
Unknown[b]	3/6	75/91	48/1166	44/47	81/1414	283/1200
**Total cases** (3924)[c]	6	91	1166	47	1414	1200

Abbreviations
WA: West African clade
Footnotes
[a] More than one route of transmission was identified as possible by authors. Possible routes: direct sexual contact, direct physical non-sexual contact fomite, inhalation.
[B] Insufficient information was reported in studies to assign or hypothesise any route of transmission
[C] Total 3924 new incident cases of mpox, reported by route of transmission.

For transmission reported within a healthcare setting, investigators considered 117/120 (97.5%) cases to either be transmitted through physical touch (non-sexual), fomite or inhalation, and 3/120 (2.5%) to be either transmitted through fomite or inhalation (Table 4). Due to limited information reported by study authors, the review team was unable to disaggregate further.

Table 4: Proportion of new incident cases of mpox by route of transmission in the healthcare setting?

<table>
<tr><td colspan="3">Population: Adults and children with confirmed mpox
Setting: Healthcare
Transmission: More than one route of transmission was identified as possible by authors. Possible routes: direct physical non- sexual contact, fomite, inhalation.</td></tr>
<tr><td>Route of transmission</td><td>Not able to be disaggregated

Number of cases/total number of cases</td><td>Partially able to be disaggregated

Number of cases/total number of cases</td></tr>
<tr><td>Physical touch (non-sexual)</td><td rowspan="3">117/120
(97.5%)</td><td>N/A</td></tr>
<tr><td>Fomite</td><td rowspan="2">3/120
(2.5%)</td></tr>
<tr><td>Inhalation</td></tr>
</table>

There were 808 cases that authors reported to have occurred within a household setting; however, due to limited reported information, no further disaggregation by route of transmission proved possible (Table 5). Authors reported possible routes of human-to-human transmission as inhalation, fomite, direct sexual physical contact and direct physical non-sexual contact.

Table 5: Proportion of new incident cases of mpox by route of transmission in the household setting

<table>
<tr><td colspan="2">Population: Adults and children with confirmed mpox
Setting: Household
Transmission: More than one route of transmission was identified as possible by authors.</td></tr>
<tr><td>Route of transmission</td><td>Number of cases/total number of cases

(Not able to be disaggregated)</td></tr>
<tr><td>Direct sexual contact</td><td rowspan="4">808/808

(100%)</td></tr>
<tr><td>Physical touch (non-sexual)</td></tr>
<tr><td>Fomite</td></tr>
<tr><td>Inhalation</td></tr>
</table>

Clinical and environmental sampling

Two studies (Hernaez 2022, Mileto 2022) attempted viral isolation from respiratory tract samples; mpox virus was isolated from saliva in 22/33 (66.7%) of samples between days 3 and 9 from symptom onset and in 1/4 (25%) oropharyngeal samples taken on day 9 from symptom onset (Table 6).

Table 6: Clinical samples of viral isolation attempts from adults or children with confirmed mpox infection

**Population:** Adults or children with confirmed mpox infection				
**Setting:** Europe, DRC, Ivory Coast, Nigeria				
Sample type	No. of samples (no. of studies)	Proportion of samples from which viral isolation successful (%)	Days on which sampling performed, (range in days from symptom onset)	Days on which viral isolation successful, range (days from symptom onset)
Saliva	33 (1)	22 of 33 (66.7%)	2 to 9	3 to 9
Oropharyngeal swabs	4 (1)	1 of 4 (25%)	9 to 12	day 9

Authors identified two studies in which viral isolation was attempted from air samples collected in rooms occupied by an individual with mpox (Hernaez 2022, Gould 2022); replication competent virus was identified in one air sample collected in a hospital room (Table 7).

Table 7: Air sampling in environments occupied by adults with confirmed mpox infection

**Study**	**Day of sampling**	**Participants**	**Clade**	**Proportion of air samples positive by PCR**	**Proportion of air samples from which virus isolated**
Hernaez 2022	2-9 post symptom onset	44	IIb	27 of 42	0 of 27
Gould 2022	6-30 post symptom onset	7	IIb	5 of 11	1 of 1[a]
**Footnotes**					
[a]Sample collected on day 9 from reported symptom onset					

Subgroups

Health care worker transmission

Health care workers were the population of interest in review questions 1 and 2.

A health care worker in the United Kingdom in 2018 was diagnosed with mpox after changing the bed linen of a confirmed mpox patient using an apron and gloves; there was no direct contact with the mpox patient (Vaughan 2018). Investigators judged that transmission was possible either by fomite or inhalational route.

Three studies reported mpox infection in a healthcare worker through percutaneous injury with a contaminated sharp object that had been in contact with an mpox lesion (Caldas 2022, Carvalho 2022, Mendoza 2022).

Patient-to-patient transmission

An outcome in review question 2 concerned the risk of transmission to patients. Two studies were identified that provided relevant data (Jezek 1986, Learned 2003). Jezek 1986 reported mpox in a

child in the Democratic Republic of the Congo who had visited a hospital several times where another child with confirmed mpox had been admitted; there was no known physical contact between the t[illegible]uthors hypothesized fomite or inhalation transmission. [illegible]ned 2003 reported a case of mpox in a patient during a hospital stay for malaria and in the same hospital as patients with mpox; authors reported no further information.

Certainty of the Evidence

For review question 1, the certainty of evidence for the outcome of mpox infection is moderate as there is no evidence comparing transmission risk between health care workers that wear a respirator to those that wear a medical mask (serious risk of indirectness; Summary of Findings Table 1). Similarly, for review question 2, the certainty of evidence for the outcome of mpox infection is moderate as there is no evidence comparing transmission risk between use of an airborne precaution room to an adequately ventilated room mask (serious risk of indirectness; Summary of Findings Table 2). Review findings indicate that inhalation is a rare route of transmission; it is inferred that there may be minimal added benefits to increased airborne infection prevention precautions.

Results: Review Question 3

Review question 3 concerned IPC interventions related to the physical isolation of patients with active lesions.

Summary of Findings Table 3: Does physical case isolation until all lesions are fully healed versus no physical case isolation until all lesions are fully healed reduce mpox infections?

Population: Adults and children with mpox
Intervention: Mpox patient does not physically isolate[a], covers all non-healed lesions, wears a medical mask.
Comparator: Mpox patient physically isolated[a] until all lesions are fully healed
Setting: Household and community settings

Outcome	Study results and measurements	Absolute effect estimates transmission		Certainty of evidence	Plain language summary
		Mpox patient isolated until all lesions are fully healed	Mpox patient does not isolate when all non-healed lesions are covered and wear a medical mask		
**Mpox infection** inferred from transmission route frequency data[b]	2420/3924 cases Direct physical sexual contact<br><br>36/3924 cases Direct physical non-sexual contact<br><br>2/3924 cases Fomite<br><br>Inferred odds ratio[c]: 1	Uncertain[d]	Uncertain[e]	Low to Moderate<br><br>Due to serious indirectness	Isolating patients probably does not prevent transmission of mpox compared to not isolating patients (provided all lesions are covered, a medical mask is worn and physical contact with others is avoided)

Footnotes
[a]Physical isolation is defined as physical separation from other people.
[b]No studies identified that directly informed the research question.
[c]Due to very minimal cases of fomite transmission, it was inferred there would be no difference between the intervention and comparator groups.
[d]The baseline transmission risk was unable to be estimated due to complete lack of data.
[e]The risk in the intervention group was unable to be estimated due to an unknown baseline risk in the comparator group.

Incident cases by route of transmission

In situations in which investigators could identify a single route of human-to-human transmission, 2420/3924 (61.7%) cases were transmitted via direct physical sexual contact, 36/3924 (0.09%) cases were transmitted via direct physical non-sexual contact, and 2/3924 (0.05%) cases via fomites (Table 2). The majority of data is from Clade IIb cases (Table 3).

The two cases transmitted via fomites occurred in health care workers who visited a patient's home for one hour, wore PPE during the visit (N95 masks, eye protection, gowns), used gloves only when taking clinical samples from patients, and did not directly physically touch the patient (Salvato 2022).

Clinical and environmental samples

Eight studies reported attempts to isolate virus from lesion samples. In four studies reporting the date of clinical sampling from symptom onset, 8/10 (80%) of lesion sample[illegible]ntained replication competent virus (Table 8) (Lapa 2022, Learned 2005, Mileto 2022, Noe 2022). In four studies in which the day of sampling was not documented, virus isolation was reported in 46.7% of lesion samples (Table 8) (Breman 1980, Erez 2019, Hughes 2021, Rimoin 2007).

Table 8: Mpox lesion clinical samples in which viral isolation was attempted

**Population:** Adults or children with confirmed mpox infection				
**Setting:** Europe, DRC, Ivory Coast, Nigeria				
Sample type	No. of samples (no. of studies)	Proportion of samples from which viral isolation successful (%)	Days on which sampling performed, range (days from symptom onset)	Days on which viral isolation successful, range (days from symptom onset)
Skin lesion swabs[a] (day of sampling reported)	10 (4)	8 of 10 (80%)	5 to 15	5 to 15
Skin lesion swabs[a] (day of sampling not reported)	199 (4)	93 of 199 (46.7%)	Not reported	Not applicable
**Footnotes**<br>[a] Skin lesion sampling includes vesicle, pustule, vesicle fluid, crusts or unspecified swab of lesion				

Five studies attempted viral isolation from environmental surface samples (Atkinson 2022, Morgan 2022, Norz 2022, Gould 2022, Pfeiffer 2022). The frequency of detection of replication competent virus was between 0 and 60% in surface samples (Table 9).

Table 9: Surface Sampling in environments occupied by adults with confirmed mpox infection

Study	Setting	Number of Participants	Clade	Day of sampling	Proportion positive by viral isolation (%)
Atkinson 2022	Household	1	IIa	3 days after patient left	6 of 10 (60%)
Morgan 2022	Household	1	IIa	15 days after patient left	7 of 31 (23%)
Norz 2022	Healthcare	2	IIb	Day 4 of occupation	3 of 40 (8%)
Gould 2022	Healthcare	7	IIb	Day 6-30 after symptom onset	1 of 3 (33%)
Pfeiffer 2022	Household	2	IIb	Day 20 of isolation (ongoing symptoms)	0 of 21 (0%)

Certainty of the Evidence

The certainty of evidence for the outcome of mpox infection (Summary of Findings Table 3) is low to moderate as there is no evidence comparing transmission risk between mpox patients that isolate to those that do not isolate (serious risk of indirectness). Review findings indicate that direct contact is the primary mode of mpox transmission; it is inferred that there may be minimal added benefits to physically isolating cases provided all lesions are covered and a medical mask is worn.

Discussion

There is no evidence from randomized controlled trials or observational comparative studies concerning respiratory interventions or case physical isolation measures in mpox or mpox-like viruses capable of human-to-human transmission. Investigators reported zero cases of instances of respiratory (inhalation) transmission of mpox as a single route of transmission. Investigators reported 2 out of 3924 (0.05%) cases in which mpox virus could have reasonably been exclusively transmitted through fomites (Salvato 2022). In comparison, in 2420/3924 (61.7%) cases investigators identified transmission occurring through direct physical sexual contact.

Study investigators identified infectious mpox virus in saliva (Hernaez 2022) and oropharyngeal swabs (Mileto 2022) and identified competent virus in 1/28 (3.6%) air samples (Hernaez 2022, Gould 2022). Viral isolation was successful in 101/209 (48%) of lesion samples from 8 studies; surface sampling in domestic and healthcare environments in 5 studies detected viable mpox virus in 16.2% of samples (range 0-60%). The presence of infectious virus in clinical samples and environmental samples provides only very low certainty evidence regarding risk of transmission.

There is scarce data concerning transmission of mpox to health care workers. Transmission of mpox to healthcare workers was identified through percutaneous injury in three cases (Caldas 2022, Carvalho 2022, Mendoza 2022) and through possible fomite or inhalation in one case (Vaughan 2018).

Strengths and limitations

This review is strengthened by a comprehensive search strategy and authors independently assessed all studies for eligibility in duplicate to identify all possible relevant literature. Further, a clear conceptual data framework to address the research questions was undertaken; key data from all available literature that could inform the research questions was identified and synthesized.

This review is limited by the existing available evidence base on mpox. The review team utilized only broad search terms inclusive of terms for mpox-like viruses however no comparative interventional studies were identified. There is limited epidemiological evidence on the risk of fomite and inhalational transmission, and limited evidence on the infectious period for different routes of transmission.

To our knowledge there is no known prior systematic review investigating respiratory or case isolation infection prevention control for mpox or mpox-like viruses.

Implications for practice and research

The findings of this review provide compelling evidence that transmission of mpox occurs primarily through direct physical contact. Epidemiological evidence and data from clinical and environmental sampling does not support the hypothesis that respiratory or fomite modes of transmission are significant. The impact of respiratory infection prevention control measures in reducing transmission may be small (moderate certainty evidence). There is potential for shedding of infectious virus onto surfaces from lesions of detached scabs; covering mpox lesions is likely to reduce onward transmission however there is probably minimal reduction in transmission from added physical isolation of patients (moderate certainty evidence). Multiple factors such as route of exposure, infecting dose, susceptibility of the exposed individual would affect the relative risk of transmission. Currently, recommendations for respiratory and case isolation infection prevention control measures in mpox rely on expert opinion and inferences from data concerning transmission frequency by route of transmission.

Conclusion

No available evidence from comparative interventional studies addressing respiratory and case isolation infection prevention control measures to prevent the transmission of mpox exists. Current findings suggest that transmission occurs primarily through direct physical contact. No cases of transmission via inhalation were identified; the impact of respiratory infection prevention control measures in reducing transmission may be minimal. Covering mpox lesions, wearing a medical mask and avoiding physical contact with others is likely to reduce onward transmission; there is probably minimal additional reduction in transmission from also physically isolating patients. Further research is needed into effective infection prevention control measures to reduce the transmission of mpox.

References

Studies included in the systematic review

1. Adler H, Gould S, Hine P, Snell LB, Wong W, Houlihan CF, et al; NHS England High Consequence Infectious Diseases (Airborne) Network. Clinical features and management of human monkeypox: a retrospective observational study in the UK. Lancet Infectious Diseases. 2022;22(8):1153-62. doi: 10.1016/S1473-3099(22)00228-6.
2. Alpalhão M, Frade JV, Sousa D, Patrocínio J, Garrido PM, Correia C, et al. Monkeypox: a new (sexually transmissible) epidemic? Journal of the European Academy of Dermatology and Venereology. 2022;36(12):e1016-7. doi: 10.1111/jdv.18424.
3. Anderson MG, Frenkel LD, Homann S, Guffey J. A case of severe monkeypox virus disease in an American child: emerging infections and changing professional values. Pediatric Infectious Disease Journal. 2003;22(12):1093-6; discussion 1096-8. doi: 10.1097/01.inf.0000101821.61387.a5.
4. Antinori A, Mazzotta V, Vita S, Carletti F, Tacconi D, Lapini LE, et al; INMI Monkeypox Group. Epidemiological, clinical and virological characteristics of four cases of monkeypox support transmission through sexual contact, Italy, May 2022. Euro Surveillance. 2022;27(22):2200421. doi: 10.2807/1560-7917.ES.2022.27.22.2200421.
6. Atkinson B, Burton C, Pottage T, Thompson KA, Ngabo D, Crook A, et al. Infection-competent monkeypox virus contamination identified in domestic settings following an imported case of monkeypox into the UK. Environmental Microbiology. 2022;24(10):4561-9. doi: 10.1111/1462-2920.16129.
7. Benatti SV, Venturelli S, Comi N, Borghi F, Paolucci S, Baldanti F. Ophthalmic manifestation of monkeypox infection. Lancet Infectious Diseases. 2022;22(9):1397. doi: 10.1016/S1473-3099(22)00504-7.
8. Besombes C, Gonofio E, Konamna X, Selekon B, Grant R, Gessain A, et al. Intrafamily transmission of monkeypox virus, Central African Republic, 2018. Emerging Infectious Diseases. 2019;25(8):1602-4. doi: 10.3201/eid2508.190112.
9. Bížová B, Veselý D, Trojánek M, Rob F. Coinfection of syphilis and monkeypox in HIV positive man in Prague, Czech Republic. Travel Medicine and Infectious Disease. 2022;49:102368. doi: 10.1016/j.tmaid.2022.102368.
10. Bociąga-Jasik M, Raczyńska A, Lara M, Kalinowska-Nowak A, Garlicki A. Monkeypox presenting with genital ulcers: a challenging clinical problem. Polish Archives of Internal Medicine. 2022;132(10):16304. doi: 10.20452/pamw.16304.
11. Breman JG, Kalisa-Ruti, Steniowski MV, Zanotto E, Gromyko AI, Arita I. Human monkeypox, 1970-79. Bulletin of the World Health Organization. 1980;58(2):165-82.
12. Brito Caldeira M, Fernandes C. Cutaneous lesions from monkeypox infection. Sexually Transmitted Diseases. 2022;49(9):595. doi: 10.1097/OLQ.0000000000001674.
13. Brundu M, Marinello S, Scaglione V, Ferrari A, Franchin E, Mazzitelli M, et al. The first case of monkeypox virus and acute HIV infection: should we consider monkeypox a new possible sexually transmitted infection? Journal of Dermatology. 2022. doi: 10.1111/1346-8138.16556.
14. Bruno G, Fabrizio C, Rodano L, Buccoliero GB. Monkeypox in a 71-year-old woman. Journal of Medical Virology. 2023;95(1):e27993. doi: 10.1002/jmv.27993.
15. Caldas JP, Valdoleiros SR, Rebelo S, Tavares M. Monkeypox after occupational needlestick injury from pustule. Emerging Infectious Diseases. 2022;28(12):2516-9. doi: 10.3201/eid2812.221374.

16. Carannante N, Tiberio C, Bellopede R, Liguori M, Di Martino F, Maturo N, et al. Monkeypox clinical features and differential diagnosis: first case in Campania Region. Pathogens. 2022;11(8):869. doi: 10.3390/pathogens11080869.
17. Carvalho LB, Casadio LVB, Polly M, Nastri AC, Turdo AC, de Araujo Eliodoro RH, et al. Monkeypox virus transmission to healthcare worker through needlestick injury, Brazil. Emerging Infectious Diseases. 2022;28(11):2334-6. doi: 10.3201/eid2811.221323.
18. Centers for Disease Control and Prevention. Human monkeypox--Kasai Oriental, Democratic Republic of Congo, February 1996-October 1997. JAMA. 1998;279(3):189-90.
19. Costello V, Sowash M, Gaur A, Cardis M, Pasieka H, Wortmann G, et al. Imported monkeypox from international traveler, Maryland, USA, 2021. Emerging Infectious Diseases. 2022;28(5):1002-5. doi: 10.3201/eid2805.220292.
20. Croft DR, Sotir MJ, Williams CJ, Kazmierczak JJ, Wegner MV, Rausch D, et al. Occupational risks during a monkeypox outbreak, Wisconsin, 2003. Emerging Infectious Diseases. 2007;13(8):1150-7. doi: 10.3201/eid1308.061365.
21. Damon IK, Roth CE, Chowdhary V. Discovery of monkeypox in Sudan. New England Journal of Medicine. 2006;355(9):962-3. doi: 10.1056/NEJMc060792.
22. Davido B, D'Anglejan E, Baudoin R, Dahmane L, Chaud A, Cortier M, et al. Monkeypox outbreak 2022: an unusual case of peritonsillar abscess in a person previously vaccinated against smallpox. Journal of Travel Medicine. 2022;29(6):taac082. doi: 10.1093/jtm/taac082.
23. De Baetselier I, Van Dijck C, Kenyon C, Coppens J, Michiels J, de Block T, et al; ITM Monkeypox study group. Retrospective detection of asymptomatic monkeypox virus infections among male sexual health clinic attendees in Belgium. Nature Medicine. 2022;28(11):2288-92. doi: 10.1038/s41591-022-02004-w.
24. de Nicolas-Ruanes B, Vivancos MJ, Azcarraga-Llobet C, Moreno AM, Rodriguez-Dominguez M, Berna-Rico ED, et al. Monkeypox virus case with maculopapular exanthem and proctitis during the Spanish outbreak in 2022. Journal of European Academy of Dermatology and Venereology. 2022;36(8):e658-60. doi: 10.1111/jdv.18300.
25. de Sousa D, Patrocínio J, Frade J, Correia C, Borges-Costa J, Filipe P. Human monkeypox coinfection with acute HIV: an exuberant presentation. International Journal of STD & AIDS. 2022;33(10):936-8. doi: 10.1177/09564624221114998.
26. Doshi RH, Guagliardo SAJ, Doty JB, Babeaux AD, Matheny A, Burgado J, et al. Epidemiologic and ecologic investigations of monkeypox, Likouala Department, Republic of the Congo, 2017. Emerging Infectious Diseases. 2019;25(2):281-9. doi: 10.3201/eid2502.181222.
27. Eltvedt AK, Christiansen M, Poulsen A. A case report of monkeypox in a 4-year-old boy from the DR Congo: challenges of diagnosis and management. Case Reports in Pediatrics. 2020;2020:8572596. doi: 10.1155/2020/8572596.
28. Erez N, Achdout H, Milrot E, Schwartz Y, Wiener-Well Y, Paran N, et al. Diagnosis of imported monkeypox, Israel, 2018. Emerging Infectious Diseases. 2019;25(5):980-3. doi: 10.3201/eid2505.190076.
29. Escudero-Tornero R, Sobral-Costas TG, De Moraes-Souza R. Monkeypox lesions affecting the nose: a therapeutical challenge. Journal of the European Academy of Dermatology and Venereology. 2022 [online ahead of print]. doi: 10.1111/jdv.18540.
30. Escudero-Tornero R, Sobral-Costas TG, Servera-Negre G. Monkeypox infection. JAMA Dermatology. 2022;158(10):1203. doi: 10.1001/jamadermatol.2022.3975.
31. European Centre for Disease Prevention and Control. Rapid risk assessment: monkeypox cases in the UK imported by travellers returning from Nigeria, 21 September 2018.

www.ecdc.europa.eu/sites/default/files/documents/13-09-2018-RRA-Monkeypox-Nigeria-United-Kingdom.pdf (accessed dd Month yyyy).

32. Ferraro F, Caraglia A, Rapiti A, Cereda D, Vairo F, Mattei G, et al. Letter to the editor: multiple introductions of MPX in Italy from different geographic areas. Euro Surveillance. 2022;27(23):2200456. doi: 10.2807/1560-7917.ES.2022.27.23.2200456.
33. Ferré VM, Bachelard A, Zaidi M, Armand-Lefevre L, Descamps D, Charpentier C, et al. Detection of monkeypox virus in anorectal swabs from asymptomatic men who have sex with men in a sexually transmitted infection screening program in Paris, France. Annals of Internal Medicine. 2022;175(10):1491-2. doi: 10.7326/M22-2183.
34. Fleischauer AT, Kile JC, Davidson M, Fischer M, Karem KL, Teclaw R, et al. Evaluation of human-to-human transmission of monkeypox from infected patients to health care workers. Clinical Infectious Diseases. 2005;40(5):689-94. doi: 10.1086/427805.
35. Formenty P, Muntasir MO, Damon I, Chowdhary V, Opoka ML, Monimart C, et al. Human monkeypox outbreak caused by novel virus belonging to Congo Basin clade, Sudan, 2005. Emerging Infectious Diseases. 2010;16(10):1539-45. doi: 10.3201/eid1610.100713.
36. Girometti N, Byrne R, Bracchi M, Heskin J, McOwan A, Tittle V, et al. Demographic and clinical characteristics of confirmed human monkeypox virus cases in individuals attending a sexual health centre in London, UK: an observational analysis. Lancet Infectious Diseases. 2022;22(9):1321-8. doi: 10.1016/S1473-3099(22)00411-X.
37. Gould S, Atkinson B, Onianwa O, Spencer A, Furneaux J, Grieves J, et al; NHS England Airborne High Consequence Infectious Diseases Network. Air and surface sampling for monkeypox virus in a UK hospital: an observational study. Lancet Microbe. 2022;3(12):e904-11. doi: 10.1016/S2666-5247(22)00257-9.
38. Griffiths-Acha J, Vela-Ganuza M, Sarró-Fuente C, López-Estebaranz JL. Monkeypox: a new differential diagnosis when addressing genital ulcer disease. British Journal of Dermatology. 2022;187(6):1050-2. doi: 10.1111/bjd.21834.
39. Hammarlund E, Lewis MW, Carter SV, Amanna I, Hansen SG, Strelow LI, et al. Multiple diagnostic techniques identify previously vaccinated individuals with protective immunity against monkeypox. Nature Medicine. 2005;11(9):1005-11. doi: 10.1038/nm1273.
40. Hammerschlag Y, MacLeod G, Papadakis G, Adan Sanchez A, Druce J, Taiaroa G, et al. Monkeypox infection presenting as genital rash, Australia, May 2022. Euro Surveillance. 2022;27(22):2200411. doi: 10.2807/1560-7917.ES.2022.27.22.2200411.
41. Hernaez B, Muñoz-Gómez A, Sanchiz A, Orviz E, Valls-Carbo A, Sagastagoitia I, et al. Monitoring monkeypox virus in saliva and air samples in Spain: a cross-sectional study. Lancet Microbe. 2023;4(1):e21-8. doi: 10.1016/S2666-5247(22)00291-9.
42. Heskin J, Belfield A, Milne C, Brown N, Walters Y, Scott C, et al. Transmission of monkeypox virus through sexual contact - a novel route of infection. Journal of Infection. 2022;85(3):334-63. doi: 10.1016/j.jinf.2022.05.028.
43. Hobson G, Adamson J, Adler H, Firth R, Gould S, Houlihan C, et al. Family cluster of three cases of monkeypox imported from Nigeria to the United Kingdom, May 2021. Euro Surveillance. 2021;26(32):2100745. doi: 10.2807/1560-7917.ES.2021.26.32.2100745. Hughes CM, Liu L, Davidson WB, Radford KW, Wilkins K, Monroe B, et al. A tale of two viruses: coinfections of monkeypox and Varicella Zoster virus in the Democratic Republic of Congo. American Journal of Tropical Medicine and Hygiene. 2020;104(2):604-11. doi: 10.4269/ajtmh.20-0589.
44. Huhn GD, Bauer AM, Yorita K, Graham MB, Sejvar J, Likos A, et al. Clinical characteristics of human monkeypox, and risk factors for severe disease. Clinical Infectious Diseases. 2005;41(12):1742-51. doi: 10.1086/498115.

45. Hutin YJ, Williams RJ, Malfait P, Pebody R, Loparev VN, Ropp SL, et al. Outbreak of human monkeypox, Democratic Republic of Congo, 1996 to 1997. Emerging Infectious Diseases. 2001;7(3):434-8. doi: 10.3201/eid0703.010311.
46. Iñigo Martínez J, Gil Montalbán E, Jiménez Bueno S, Martín Martínez F, Nieto Juliá A, Sánchez Díaz J, et al. Monkeypox outbreak predominantly affecting men who have sex with men, Madrid, Spain, 26 April to 16 June 2022. Euro Surveillance. 2022;27(27):2200471. doi: 10.2807/1560-7917.ES.2022.27.27.2200471.
47. Jang YR, Lee M, Shin H, Kim JW, Choi MM, Kim YM, et al. The first case of monkeypox in the Republic of Korea. Journal of Korean Medical Science. 2022;37(27):e224. doi: 10.3346/jkms.2022.37.e224.
48. Jezek Z, Arita I, Mutombo M, Dunn C, Nakano JH, Szczeniowski M. Four generations of probable person-to-person transmission of human monkeypox. American Journal of Epidemiology. 1986;123(6):1004-12. doi: 10.1093/oxfordjournals.aje.a114328.
49. Jezek Z, Grab B, Szczeniowski M, Paluku KM, Mutombo M. Clinico-epidemiological features of monkeypox patients with an animal or human source of infection. Bulletin of the World Health Organization. 1988;66(4):459-64.
50. Jezek Z, Marennikova SS, Mutumbo M, Nakano JH, Paluku KM, Szczeniowski M. Human monkeypox: a study of 2,510 contacts of 214 patients. Journal of Infectious Diseases. 1986;154(4):551-5. doi: 10.1093/infdis/154.4.551.
51. Kalthan E, Tenguere J, Ndjapou SG, Koyazengbe TA, Mbomba J, Marada RM, et al. Investigation of an outbreak of monkeypox in an area occupied by armed groups, Central African Republic. Medicines et Maladies Infectieuses. 2018 Jun;48(4):263-8. doi: 10.1016/j.medmal.2018.02.010.
52. Karan A, Styczynski AR, Huang C, Sahoo MK, Srinivasan K, Pinsky BA, et al. Human monkeypox without viral prodrome or sexual exposure, California, USA, 2022. Emerging Infectious Diseases. 2022;28(10):2121-3. doi: 10.3201/eid2810.221191.
53. Karem KL, Reynolds M, Hughes C, Braden Z, Nigam P, Crotty S, et al. Monkeypox-induced immunity and failure of childhood smallpox vaccination to provide complete protection. Clinical and Vaccine Immunology. 2007;14(10):1318-27. doi: 10.1128/CVI.00148-07.
54. Kawsar A, Hussain K, Roberts N. Monkeypox: key pointers for dermatologists. Clinical and Experimental Dermatology. 2022 Dec;47(12):2282-4. doi: 10.1111/ced.15357.
55. Kile JC, Fleischauer AT, Beard B, Kuehnert MJ, Kanwal RS, Pontones P, et al. Transmission of monkeypox among persons exposed to infected prairie dogs in Indiana in 2003. Archives of Pediatrics & Adolescent Medicine. 2005;159(11):1022-5. doi: 10.1001/archpedi.159.11.1022.
56. Koh XQ, Chio MTW, Tan M, Leo YS, Chan RKW. Global monkeypox outbreak 2022: first case series in Singapore. Annals of the Academy of Medicine, Singapore. 2022;51(8):462-72. doi: 10.47102/annals-acadmedsg.2022269.
57. Kreuter A, Grossmann M, Müller VL, Michalowitz AL, Oellig F, Silling S, et al. Monkeypox infection with concomitant penile lymphoedema. Journal of the European Academy of Dermatology and Venereology. 2022. doi: 10.1111/jdv.18502. Epub ahead of print.
58. Lapa D, Carletti F, Mazzotta V, Matusali G, Pinnetti C, Meschi S, et al; INMI Monkeypox Study Group. Monkeypox virus isolation from a semen sample collected in the early phase of infection in a patient with prolonged seminal viral shedding. Lancet Infectious Diseases. 2022;22(9):1267-9. doi: 10.1016/S1473-3099(22)00513-8.
59. Learned LA, Reynolds MG, Wassa DW, Li Y, Olson VA, Karem K, et al. Extended interhuman transmission of monkeypox in a hospital community in the Republic of the Congo, 2003. American Journal of Tropical Medicine and Hygiene. 2005;73(2):428-34.

60. Lewis A, Josiowicz A, Hirmas Riade SM, Tous M, Palacios G, Cisterna DM. Introduction and differential diagnosis of monkeypox in Argentina, 2022. Emerging Infectious Diseases. 2022;28(10):2123-5. doi: 10.3201/eid2810.221075.
61. Lucar J, Roberts A, Saardi KM, Yee R, Siegel MO, Palmore TN. Monkeypox virus-associated severe proctitis treated with oral tecovirimat: a report of two cases. Annals of Internal Medicine. 2022;175(11):1626-7. doi: 10.7326/L22-0300.
62. Mailhe M, Beaumont AL, Thy M, Le Pluart D, Perrineau S, Houhou-Fidouh N, et al. Clinical characteristics of ambulatory and hospitalized patients with monkeypox virus infection: an observational cohort study. Clinical Microbiology and Infectious Diseases. 2022:S1198-743X(22)00428-1. doi: 10.1016/j.cmi.2022.08.012. Epub ahead of print.
63. Mande G, Akonda I, De Weggheleire A, Brosius I, Liesenborghs L, Bottieau E, et al. Enhanced surveillance of monkeypox in Bas-Uélé, Democratic Republic of Congo: the limitations of symptom-based case definitions. International Journal of Infectious Diseases. 2022;122:647-55. doi: 10.1016/j.ijid.2022.06.060.
64. Maronese CA, Beretta A, Avallone G, Boggio FL, Marletta DA, Murgia G, et al. Clinical, dermoscopic and histopathological findings in localized human monkeypox: a case from northern Italy. British Journal of Dermatology. 2022;187(5):822-3. doi: 10.1111/bjd.21773.
65. Mathieson T, Dulguerov N, Mermod M. Monkeypox mucosal lesions. IDCases. 2022;29:e01600. doi: 10.1016/j.idcr.2022.e01600.
66. Mazzotta V, Mondi A, Carletti F, Baldini F, Santoro R, Meschi S, et al. Ocular involvement in monkeypox: description of an unusual presentation during the current outbreak. Journal of Infection. 2022;85(5):573-607. doi: 10.1016/j.jinf.2022.08.011.
67. Mbala PK, Huggins JW, Riu-Rovira T, Ahuka SM, Mulembakani P, Rimoin AW, et al. Maternal and fetal outcomes among pregnant women with human monkeypox infection in the Democratic Republic of Congo. Journal of Infectious Diseases. 2017;216(7):824-8. doi: 10.1093/infdis/jix260.
68. Mendoza R, Petras JK, Jenkins P, Gorensek MJ, Mableson S, Lee PA, et al. Monkeypox virus infection resulting from an occupational needlestick – Florida, 2022. MMWR Morbidity and Mortality Weekly Report. 2022;71(42):1348-9. doi: 10.15585/mmwr.mm7142e2.
69. Menezes YR, Miranda AB. Severe disseminated clinical presentation of monkeypox virus infection in an immunosuppressed patient: first death report in Brazil. Revista da Sociedade Brasileira de Medicina Tropical. 2022;55:e0392. doi: 10.1590/0037-8682-0392-2022.
70. Mileto D, Riva A, Cutrera M, Moschese D, Mancon A, Meroni L, et al. New challenges in human monkeypox outside Africa: a review and case report from Italy. Travel Medicine and Infectious Disease. 2022;49:102386. doi: 10.1016/j.tmaid.2022.102386.
71. Morgan CN, Whitehill F, Doty JB, Schulte J, Matheny A, Stringer J, et al. Environmental persistence of monkeypox virus on surfaces in household of person with travel-associated infection, Dallas, Texas, USA, 2021. Emerging Infectious Diseases. 2022;28(10):1982-9. doi: 10.3201/eid2810.221047.
72. Müller G, Meyer A, Gras F, Emmerich P, Kolakowski T, Esposito JJ. Monkeypox virus in liver and spleen of child in Gabon. Lancet. 1988;1(8588):769-70. doi: 10.1016/s0140-6736(88)91580-2.
73. Nakoune E, Lampaert E, Ndjapou SG, Janssens C, Zuniga I, Van Herp M, et al. A nosocomial outbreak of human monkeypox in the Central African Republic. Open Forum Infectious Diseases. 2017;4(4):ofx168. doi: 10.1093/ofid/ofx168.
74. Ng OT, Lee V, Marimuthu K, Vasoo S, Chan G, Lin RTP, Leo YS. A case of imported monkeypox in Singapore. Lancet Infectious Diseases. 2019 Nov;19(11):1166. doi: 10.1016/S1473-3099(19)30537-7.

75. Noe S, Zange S, Seilmaier M, Antwerpen MH, Fenzl T, Schneider J, et al. Clinical and virological features of first human monkeypox cases in Germany. Infection. 2023;51(1):265-70. doi: 10.1007/s15010-022-01874-z.
76. Nolasco S, Vitale F, Geremia A, Tramuto F, Maida CM, Sciuto A, et al. First case of monkeypox virus, SARS-CoV-2 and HIV co-infection. Journal of Infection. 2023;86(1):e21-3. doi: 10.1016/j.jinf.2022.08.014.
77. Nolen LD, Osadebe L, Katomba J, Likofata J, Mukadi D, Monroe B, et al. Extended human-to-human transmission during a monkeypox outbreak in the Democratic Republic of the Congo. Emerging Infectious Diseases. 2016;22(6):1014-21. doi: 10.3201/eid2206.150579.
78. Nolen LD, Osadebe L, Katomba J, Likofata J, Mukadi D, Monroe B, et al. Introduction of monkeypox into a community and household: risk factors and zoonotic reservoirs in the Democratic Republic of the Congo. American Journal of Tropical Medicine and Hygiene. 2015;93(2):410-5. doi: 10.4269/ajtmh.15-0168.
79. Nörz D, Pfefferle S, Brehm TT, Franke G, Grewe I, Knobling B, et al. Evidence of surface contamination in hospital rooms occupied by patients infected with monkeypox, Germany, June 2022. Euro Surveillance. 2022;27(26):2200477. doi: 10.2807/1560-7917.ES.2022.27.26.2200477.
80. Ogoina D, Yinka-Ogunleye A. Sexual history of human monkeypox patients seen at a tertiary hospital in Bayelsa, Nigeria. International Journal of STD & AIDS. 2022;33(10):928-32. doi: 10.1177/09564624221119335.
81. Ogoina D, Izibewule JH, Ogunleye A, Ederiane E, Anebonam U, Neni A, et al. The 2017 human monkeypox outbreak in Nigeria-report of outbreak experience and response in the Niger Delta University Teaching Hospital, Bayelsa State, Nigeria. PLoS One. 2019;14(4):e0214229. doi: 10.1371/journal.pone.0214229.
82. Oprea C, Ianache I, Piscu S, Tardei G, Nica M, Ceausu E, et al. First report of monkeypox in a patient living with HIV from Romania. Travel Medicine and Infectious Diseases. 2022;49:102395. doi: 10.1016/j.tmaid.2022.102395.
83. Ortiz-Martínez Y, Rodríguez-Morales AJ, Franco-Paredes C, Chastain DB, Gharamti AA, Vargas Barahona L, et al. Monkeypox - a description of the clinical progression of skin lesions: a case report from Colorado, USA. Therapeutic Advances in Infectious Disease. 2022;9:20499361221117726. doi: 10.1177/20499361221117726.
84. Orviz E, Negredo A, Ayerdi O, Vázquez A, Muñoz-Gomez A, Monzón S, et al; Grupo Viruela del Simio Madrid CNM/ISCIII/HCSC/Sandoval. Monkeypox outbreak in Madrid (Spain): Clinical and virological aspects. Journal of Infection. 2022;85(4):412-7. doi: 10.1016/j.jinf.2022.07.005.
85. Paparizos V, Nicolaidou E, Tryfinopoulou K, Papa A, Rigopoulos D, Tsiodras S, Stratigos A. Monkeypox virus infection: First reported case in Greece in a patient with a genital rash. Journal of the European Academy of Dermatology and Venereology. 2022 Aug 14. doi: 10.1111/jdv.18521. Epub ahead of print.
86. Paran N, Yahalom-Ronen Y, Shifman O, Lazar S, Ben-Ami R, Yakubovsky M, Levy I, Wieder-Feinsod A, Amit S, Katzir M, Carmi-Oren N, Levcovich A, Hershman-Sarafov M, Paz A, Thomas R, Tamir H, Cherry-Mimran L, Erez N, Melamed S, Barlev-Gross M, Karmi S, Politi B, Achdout H, Weiss S, Levy H, Schuster O, Beth-Din A, Israely T. Monkeypox DNA levels correlate with virus infectivity in clinical samples. Eurosurveillance. 2022;27(35):pii=2200636. https://doi.org/10.2807/1560-7917.ES.2022.27.35.2200636
87. Patel A, Bilinska J, Tam JCH, Da Silva Fontoura D, Mason CY, Daunt A, et al. Clinical features and novel presentations of human monkeypox in a central London centre during the 2022

outbreak: descriptive case series. BMJ. 2022 Jul 28;378:e072410. doi: 10.1136/bmj-2022-072410.

88. Patrocinio-Jesus R, Peruzzu F. Monkeypox Genital Lesions. New England Journal of Medicine. 2022 Jul 7;387(1):66. doi: 10.1056/NEJMicm2206893.
89. Peiró-Mestres A, Fuertes I, Camprubí-Ferrer D, Marcos MÁ, Vilella A, Navarro M, et al; Hospital Clinic de Barcelona Monkeypox Study Group. Frequent detection of monkeypox virus DNA in saliva, semen, and other clinical samples from 12 patients, Barcelona, Spain, May to June 2022. Euro Surveillance. 2022;27(28):2200503. doi: 10.2807/1560-7917.ES.2022.27.28.2200503.
90. Pembi E, Awang S, Salaudeen SO, Agaba IA, Omoleke S. First confirmed case of monkeypox in Adamawa State, Nigeria: a clinico-epidemiological case report. Pan African Medical Journal. 2022;42:38. doi: 10.11604/pamj.2022.42.38.34715.
91. Perez Duque M, Ribeiro S, Martins JV, Casaca P, Leite PP, Tavares M, et al. Ongoing monkeypox virus outbreak, Portugal, 29 April to 23 May 2022. Euro Surveillance. 2022;27(22):2200424. doi: 10.2807/1560-7917.ES.2022.27.22.2200424.
92. Pfäfflin F, Wendisch D, Scherer R, Jürgens L, Godzick-Njomgang G, Tranter E, et al. Monkeypox in-patients with severe anal pain. Infection. 2022. doi: 10.1007/s15010-022-01896-7. Epub ahead of print.
93. Pfeiffer JA, Collingwood A, Rider LE, Minhaj FS, Matheny AM, Kling C, et al. High-contact object and surface contamination in a household of persons with monkeypox virus infection - Utah, June 2022. MMWR Morbidity and Mortality Weekly Report. 2022;71(34):1092-4. doi: 10.15585/mmwr.mm7134e1.
94. Philpott D, Hughes CM, Alroy KA, Kerins JL, Pavlick J, Asbel L, et a; CDC Multinational Monkeypox Response Team. Epidemiologic and clinical characteristics of monkeypox cases - United States, May 17-July 22, 2022. MMWR Morbidity and Mortality Weekly Report. 2022;71(32):1018-22. doi: 10.15585/mmwr.mm7132e3.
95. Pipitò L, Cascio A. Monkeypox virus infection and creatine phosphokinase increase: a case from Italy. Travel Medicine and Infectious Diseases. 2022;50:102412. doi: 10.1016/j.tmaid.2022.102412.
96. Pittman PR, Martin JW, Kingebeni PM, Tamfum JJM, Wan Q, Reynolds MG, et al. Clinical characterization of human monkeypox infections in the Democratic Republic of the Congo. 2022. medRxiv 2022.05.26.22273379; doi: https://doi.org/10.1101/2022.05.26.22273379
97. Portela-Dias J, Sereno S, Falcão-Reis I, Rasteiro C. Monkeypox infection with localized genital lesions in women. American Journal of Obstetrics and Gynecology. 2022;227(6):906. doi: 10.1016/j.ajog.2022.08.046.
98. Quattri E, Avallone G, Maronese CA, Cusini M, Carrera CG, Marzano AV, Ramoni S. Unilesional monkeypox: a report of two cases from Italy. Travel Medicine and Infectious Diseases. 2022;49:102424. doi: 10.1016/j.tmaid.2022.102424.
99. Raccagni AR, Mileto D, Canetti D, Tamburini AM, Rizzo A, Bruzzesi E, et al. Monkeypox and pan-resistant Campylobacter spp infection in Entamoeba histolytica and Chlamydia trachomatis re-infection in a man who have sex with men. Journal of Infection. 2022;85(4):436-80. doi: 10.1016/j.jinf.2022.06.028.
100. Rao AK, Schulte J, Chen TH, Hughes CM, Davidson W, Neff JM, et al; July 2021 Monkeypox Response Team. Monkeypox in a traveler returning from Nigeria - Dallas, Texas, July 2021. MMWR Morbidity and Mortality Weekly Report. 2022;71(14):509-16. doi: 10.15585/mmwr.mm7114a1.
101. Reynolds MG, Emerson GL, Pukuta E, Karhemere S, Muyembe JJ, Bikindou A, et al. Detection of human monkeypox in the Republic of the Congo following intensive community

education. American Journal of Tropical Medicine and Hygiene. 2013;88(5):982-5. doi: 10.4269/ajtmh.12-0758.
102. Reynolds MG, Wauquier N, Li Y, Satheshkumar PS, Kanneh LD, Monroe B, et al. Human monkeypox in Sierra Leone after 44-year absence of reported cases. Emerging Infectious Diseases. 2019 May;25(5):1023-5. doi: 10.3201/eid2505.180832.
103. Rimoin AW, Kisalu N, Kebela-Ilunga B, Mukaba T, Wright LL, Formenty P, et al. Endemic human monkeypox, Democratic Republic of Congo, 2001-2004. Emerging Infectious Diseases. 2007 Jun;13(6):934-7. doi: 10.3201/eid1306.061540.
104. Sadeuh-Mba SA, Yonga MG, Els M, Batejat C, Eyangoh S, Caro V, Etoundi A, Carniel E, Njouom R. Monkeypox virus phylogenetic similarities between a human case detected in Cameroon in 2018 and the 2017-2018 outbreak in Nigeria. Infection, Genetics and Evolution. 2019;69:8-11. doi: 10.1016/j.meegid.2019.01.006.
105. Salvato RS, Rodrigues Ikeda ML, Barcellos RB, Godinho FM, Sesterheim P, Bitencourt LCB, et al. Possible occupational infection of healthcare workers with monkeypox virus, Brazil. Emerging Infectious Diseases. 2022;28(12):2520-3. doi: 10.3201/eid2812.221343.
106. Seang S, Burrel S, Todesco E, Leducq V, Monsel G, Le Pluart D, et al. Evidence of human-to-dog transmission of monkeypox virus. Lancet. 2022;400(10353):658-9. doi: 10.1016/S0140-6736(22)01487-8.
107. Suárez Rodríguez B, Guzmán Herrador BR, Díaz Franco A, Sánchez-Seco Fariñas MP, Del Amo Valero J, Aginagalde Llorente AH, et al. Epidemiologic features and control measures during monkeypox outbreak, Spain, June 2022. Emerging Infectious Diseases. 2022;28(9):1847-51. doi: 10.3201/eid2809.221051.
108. Sukhdeo SS, Aldhaheri K, Lam PW, Walmsley S. A case of human monkeypox in Canada. CMAJ. 2022;194(29):E1031-5. doi: 10.1503/cmaj.220886.
109. Tan DHS, Jaeranny S, Li M, Sukhdeo SS, Monge JC, Callejas MF, et al. Atypical clinical presentation of monkeypox complicated by myopericarditis. Open Forum Infectious Diseases. 2022;9(8):ofac394. doi: 10.1093/ofid/ofac394.
110. Tan WYT, Wong CS, Ho MZJ, Said Z, Cui L, Lin RTP, et al. The first imported case of monkeypox in Singapore during the 2022 outbreak - reflections and lessons. Travel Medicine and Infectious Diseases. 2022;50:102431. doi: 10.1016/j.tmaid.2022.102431.
111. Tarín-Vicente EJ, Alemany A, Agud-Dios M, Ubals M, Suñer C, Antón A, et al. Clinical presentation and virological assessment of confirmed human monkeypox virus cases in Spain: a prospective observational cohort study. Lancet. 2022;400(10353):661-9. doi: 10.1016/S0140-6736(22)01436-2.
112. Thornhill JP, Barkati S, Walmsley S, Rockstroh J, Antinori A, Harrison LB, et al; SHARE-net Clinical Group. Monkeypox virus infection in humans across 16 countries - April-June 2022. New England Journal of Medicine. 2022;387(8):679-91. doi: 10.1056/NEJMoa2207323.
113. Torster L, Tegtmeyer J, Kött J, Christolouka M, Schneider SW. Localized monkeypox infestation in MSM on pre-exposure prophylaxis. Journal of European Academy of Dermatology and Venereology. 2022. doi: 10.1111/jdv.18539. Epub ahead of print.
114. Turco M, Mancuso FR, Pisano L. A monkeypox virus infection mimicking primary syphilis. British Journal of Dermatology. 2022;187(6):e194-5. doi: 10.1111/bjd.21847.
115. Tutu van Furth AM, van der Kuip M, van Els AL, Fievez LC, van Rijckevorsel GG, van den Ouden A, et al. Paediatric monkeypox patient with unknown source of infection, the Netherlands, June 2022. Euro Surveillance. 2022;27(29):2200552. doi: 10.2807/1560-7917.ES.2022.27.29.2200552.

116. Vallée A, Farfour E, Zucman D. Monkeypox virus: a novel sexually transmitted disease? A case report from France. Travel Medicine and Infectious Disease. 2022;49:102394. doi: 10.1016/j.tmaid.2022.102394.
117. Vaughan A, Aarons E, Astbury J, Balasegaram S, Beadsworth M, Beck CR, et al. Two cases of monkeypox imported to the United Kingdom, September 2018. Euro Surveillance. 2018;23(38):1800509. doi: 10.2807/1560-7917.ES.2018.23.38.1800509.
118. Vaughan A, Aarons E, Astbury J, Brooks T, Chand M, Flegg P, et al. Human-to-human transmission of monkeypox virus, United Kingdom, October 2018. Emerging Infectious Diseases. 2020;26(4):782-5. doi: 10.3201/eid2604.191164.
119. Veintimilla C, Catalán P, Alonso R, de Viedma DG, Pérez-Lago L, Palomo M, et al. The relevance of multiple clinical specimens in the diagnosis of monkeypox virus, Spain, June 2022. Euro Surveillance. 2022;27(33):2200598. doi: 10.2807/1560-7917.ES.2022.27.33.2200598.
120. Vusirikala A, Charles H, Balasegaram S, Macdonald N, Kumar D, Barker-Burnside C, et al. Epidemiology of early monkeypox virus transmission in sexual networks of gay and bisexual men, England, 2022. Emerging Infectious Diseases. 2022;28(10):2082-6. doi: 10.3201/eid2810.220960.
121. Whitehouse ER, Bonwitt J, Hughes CM, Lushima RS, Likafi T, Nguete B, et al. Clinical and epidemiological findings from enhanced monkeypox surveillance in Tshuapa Province, Democratic Republic of the Congo during 2011-2015. Journal of Infectious Diseases. 2021;223(11):1870-8. doi: 10.1093/infdis/jiab133.
122. Yadav PD, Reghukumar A, Sahay RR, K S, Shete AM, Raman A, et al. First two cases of Monkeypox virus infection in travellers returned from UAE to India, July 2022. Journal of Infection. 2022;85(5):e145-8. doi: 10.1016/j.jinf.2022.08.007.
123. Yang ZS, Lin CY, Urbina AN, Wang WH, Assavalapsakul W, Tseng SP, et al. The first case of monkeypox virus infection detected in Taiwan: awareness and preparation. International Journal of Infectious Diseases. 2022;122:991-5. doi: 10.1016/j.ijid.2022.07.051.
124. Yinka-Ogunleye A, Aruna O, Dalhat M, Ogoina D, McCollum A, Disu Y, et al; CDC Monkeypox Outbreak Team. Outbreak of human monkeypox in Nigeria in 2017-18: a clinical and epidemiological report. Lancet Infectious Diseases. 2019;19(8):872-9. doi: 10.1016/S1473-3099(19)30294-4.
125. Yinka-Ogunleye A, Aruna O, Ogoina D, Aworabhi N, Eteng W, Badaru S, et al. Reemergence of human monkeypox in Nigeria, 2017. Emerging Infectious Diseases. 2018;24(6):1149-51. doi: 10.3201/eid2406.180017.
126. Yong SEF, Ng OT, Ho ZJM, Mak TM, Marimuthu K, Vasoo S, et al. Imported monkeypox, Singapore. Emerging Infectious Diseases. 2020;26(8):1826-30. doi: 10.3201/eid2608.191387.
127. Zlámal M, Bartovská Z, Burantová A, Zákoucká H, Jiřincová H, Chmel M, Holub M. Monkeypox and Herpes Simplex Virus Type 2 coinfection: case report of perianal lesions in HIV-positive patient. Sexually Transmitted Diseases. 2022;49(11):769-70. doi: 10.1097/OLQ.0000000000001694.

Other references

- DistillerSR. Version 2.35. Evidence Partners; 2021. Accessed September 2022. https://www.evidencepartners.com
- Garritty C, Gartlehner G, Kamel C, King VJ, Nussbaumer-Streit B, Stevens A, Hamel C, Affengruber L. Cochrane Rapid Reviews. Interim Guidance from the Cochrane Rapid Reviews Methods Group. March 2020.
- Schünemann HJ, Higgins JP, Vist GE, Glasziou P, Akl EA, Skoetz N, et al. Chapter 14: Completing ‘Summary of findings’ tables and grading the certainty of the evidence. In: Higgins JP, Thomas J, Chandler J, Cumpston M, Li T, Page MJ, Welch VA editor(s). Cochrane Handbook for Systematic Reviews of Interventions Version 6.3 (updated February 2022). Available from training.cochrane.org/handbook.
- WHO 2022. Surveillance, case investigation and contact tracing for monkeypox. Interim guidance. 25 August 2022. World Health Organization. WHO reference number: WHO/MPX/Surveillance/2022.3

Appendix 1. Search Strategy

Search: Monkeypox virus only

Ovid MEDLINE(R) and In-Process, In-Data-Review & Other Non-Indexed Citations

and Embase (OVID)

Date of search: 8th September 2022

1 Monkeypox virus/ or Monkeypox/

2 monkeypox.ti,ab.

3 monkey pox.mp.

4 1 or 2 or 3

Biosis previews (Web of Science) and CAB Abstracts (Web of science):

Date of search: 8th September 2022

Topic = monkeypox*

Global Index Medicus

Date of search: 26th September 2022

Search term: “monkeypox”

Search: Mpox-like viruses

Ovid MEDLINE(R) and In-Process, In-Data-Review & Other Non-Indexed Citations <1946 to September 27, 2022>

Date of search: 28th September 2022

1	orthopoxvirus/ or cowpox virus/ or ectromelia virus/ or vaccinia virus/ or variola virus/
2	(orthopox* or cowpox or vaccinia or variola or buffalopox).tw.
3	1 or 2
8	Masks/ or mask*.mp.
9	Ventilation/ or ventilation.mp.
10	air quality.mp.
11	patient isolation.mp. or Patient Isolation/
12	8 or 9 or 10 or 11
14	3 and 12
15	nosocomial transmission.mp. or Infection Control/
16	hospital transmission.mp.
17	15 or 16
18	3 and 17
19	18 or 14

Embase 1947-Present,

Date of search: 28th September 2022

1	orthopoxvirus/ or cowpox virus/ or ectromelia virus/ or vaccinia virus/ or variola virus/
2	(orthopox* or cowpox or vaccinia or variola or buffalopox).tw.
3	1 or 2
4	Masks/ or mask*.mp.
5	Ventilation/ or ventilation.mp.
6	air quality.mp.
7	patient isolation.mp. or Patient Isolation/
8	4 or 5 or 6 or 7
9	3 and 8

10 nosocomial transmission.mp. or Infection Control/

11 hospital transmission.mp.

12 10 or 11

13 3 and 12

15 9 or 13

16 monkeypox.m_titl.

17 15 not 16

Web of Science (BIOSIS Previews, CABI: CAB Abstracts®)

Date of search: 28th September 2022

#6 #4 OR #5

#5 #1 AND #3

#4 #1 AND #2

#3 "nosocomial transmission" or "Infection Control" or "hospital transmission" (Topic)

#2 mask* or Ventilation or "air quality" or "patient isolation" (Topic)

#1 orthopoxvirus* or cowpox or vaccinia or variola or buffalopox (Topic)

Global Index medicus

Date of search: 28th September 2022

tw:((tw:(orthopoxvirus* OR cowpox OR vaccinia OR variola OR buffalopox)) AND (tw:(mask* OR ventilation OR "air quality" OR "patient isolation" OR "nosocomial transmission" OR "Infection Control" OR "hospital transmission")))

Appendix 2. PRISMA flowchart

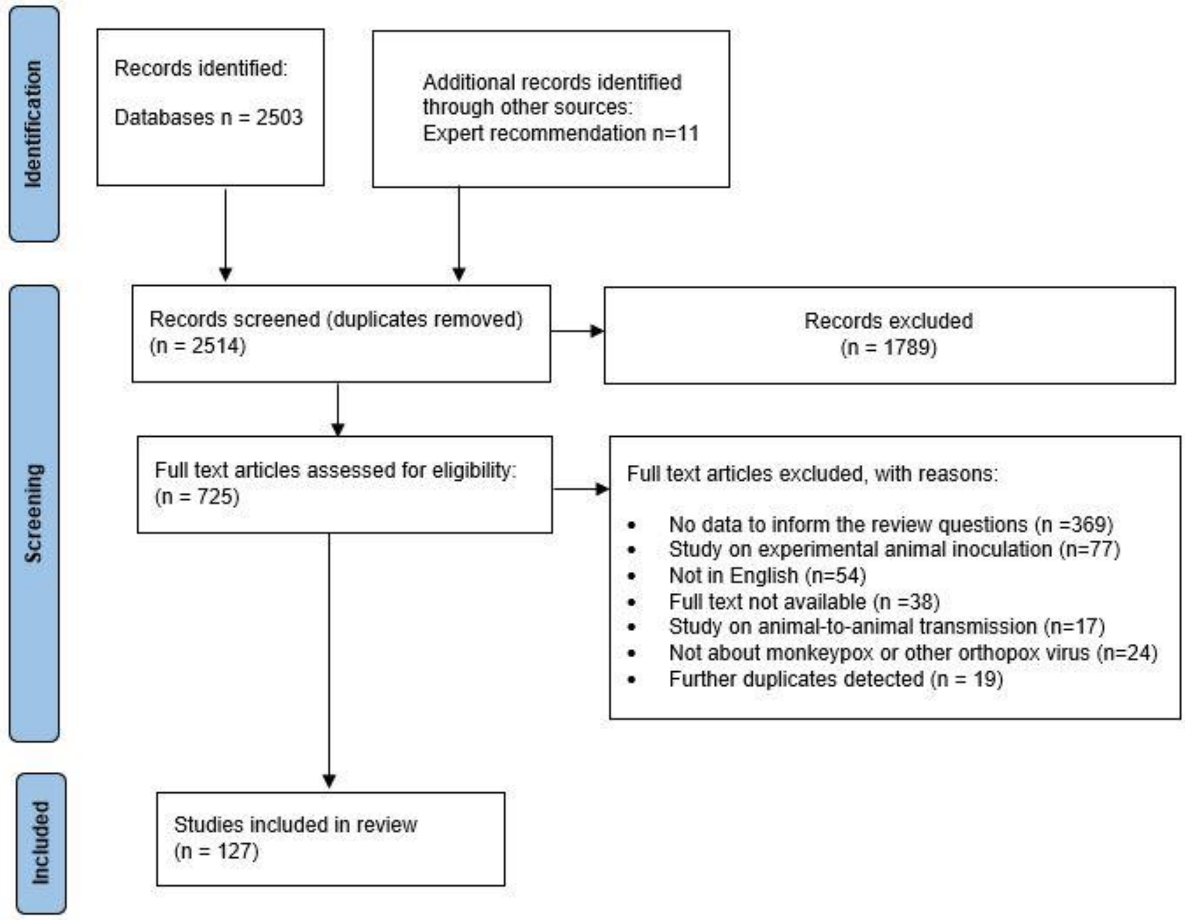

Click here to access/download;Figure;flow digram.docx

PRISMA flowchart

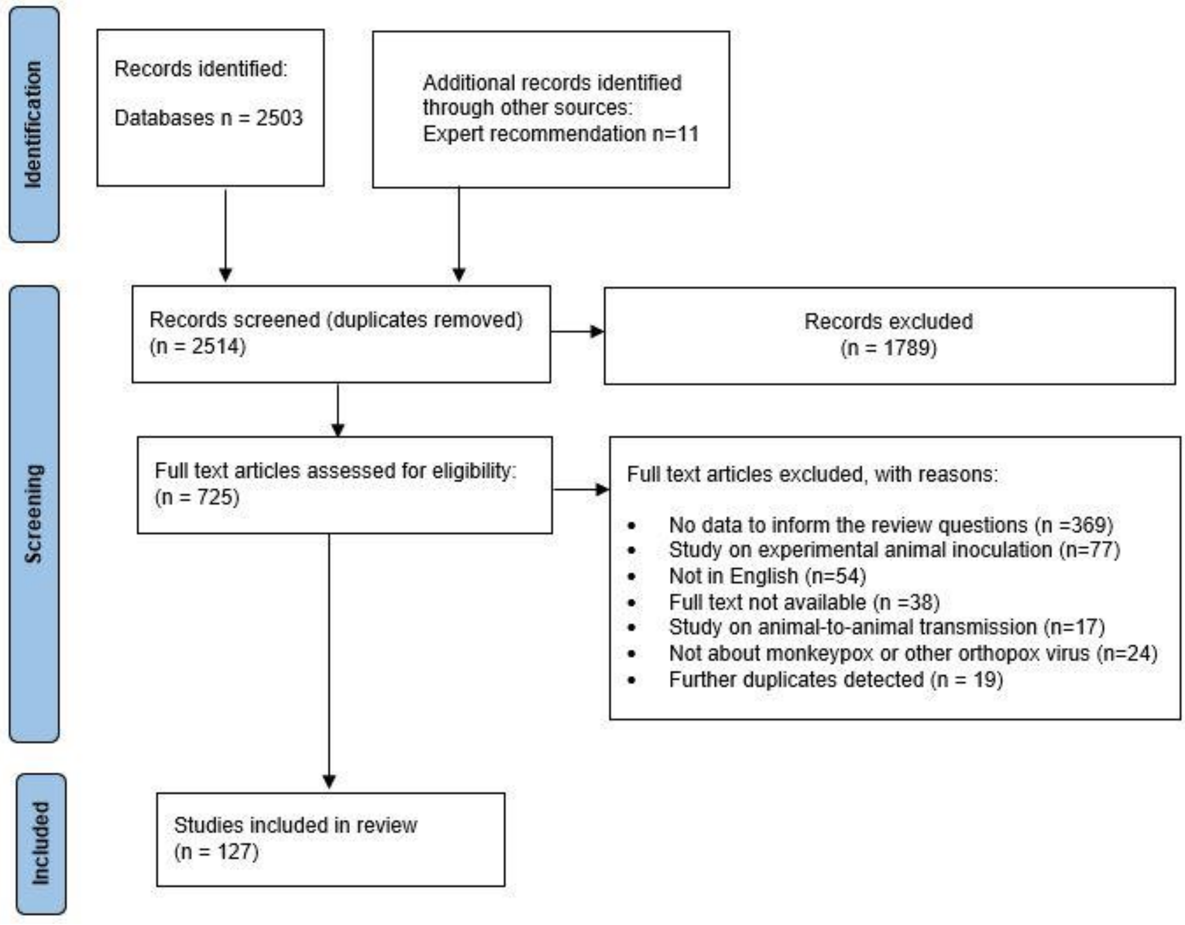

Prisma checklist

Click here to access/download
Supporting Information
PRISMA_2020_checklist.docx
