# Supplementary material for: Infection prevention and control measures to reduce the transmission of mpox: A systematic review
Source: PLOS Glob Public Health. 2024 Jan 18;4(1):e0002731. doi: 10.1371/journal.pgph.0002731 (PMC10796032; doi:10.1371/journal.pgph.0002731)
Supplement: Attachment — Submitted filename: PGPH-D-23-00439_Editor comments.docx [file pgph.0002731.s015.docx]

**Comments to the Author**

The manuscript is informative. I have added few more specific comments with reviewer that need to address.

Specific comments:

1. Abstract: Authors have mentioned the objective as “To assess the effectiveness of respiratory interventions…” As this is a review manuscript, authors have no scope to assess the effectiveness. Authors can use alternative words (e.g. review, summarize) instead of “assess”.
2. Abstract: Though the main objective was to assess the effectiveness of intervention but no information was included in result section about intervention.
3. Abstract: Transmission through inhalation is not reported as a single route. Why you suggested to wear medical mask to reduce transmission.
4. Introduction: No references are included. Why?
5. Methods: Please check the exclusion criteria. How many exclusion criteria did you consider? Any of the criteria?
6. Use reference for WHO Clinical Management and Infection Prevention and Control guideline
7. Provide a definition for “suspected case of mpox infection”.
8. Page 8: Data synthesis: “What is the proportion of new incident cases…...” Is it new cases or 2ndary cases? Why no cohort studies were considered for “What is the infectious period of mpox, disaggregated by route of transmission?”
9. Page 9: These following sentences are mentioned in method section.

“No randomized controlled trials or observational comparative studies were identified that directly informed any of the IPC review questions. No studies concerning mpox-like viruses met the inclusion criteria.” Avoid repeated information.

10. Page 10: the footnotes c and d are not properly identified
11. Page 11: the footnotes c and d are not properly identified
12. Table 3, 4 and 5: It is better to mention the study number.
13. Page 17: Inferred odds ratio^c^: 1 : Is it suggested by a study or is it your interpretation? If it its resulted from other study, please add reference.
14. Page 17: Which term is more appropriate? Incident cases or 2ndary cases? Human-human transmission is not always required for Incident cases.
15. Discussion: First paragraph: This is useful if you add some logics that support direct physical sexual contact increase the risk of mpox transmission.
16. Discussion: no discussion for suggested interventions to reduce mpox transmission. Please add a paragraph for that.
