# Supplementary material for: Infection prevention and control measures to reduce the transmission of mpox: A systematic review
Source: PLOS Glob Public Health. 2024 Jan 18;4(1):e0002731. doi: 10.1371/journal.pgph.0002731 (PMC10796032; doi:10.1371/journal.pgph.0002731)
Supplement: Attachment — Submitted filename: Response to Review Comments 29.09.2023.docx [file pgph.0002731.s016.docx]

| Reviewer comments provided in Word: | Author team response: |
| --- | --- |
| 1. Abstract: Authors have mentioned the objective as “To assess the effectiveness of respiratory interventions…” As this is a review manuscript, authors have no scope to assess the effectiveness. Authors can use alternative words (e.g. review, summarize) instead of “assess”. | Thank you, we have changed the objective in the abstract and main text as follows: To make inferences regarding the effectiveness of respiratory interventions and case isolation measures in reducing or preventing the transmission of mpox on the basis of synthesis of available literature. |
| 2. Abstract: Though the main objective was to assess the effectiveness of intervention but no information was included in result section about intervention. | Thank you for this comment.  We have added the following statement in the Results section to clarify the finding of no direct studies addressing the interventions: “No studies were identified that directly addressed respiratory and isolation infection prevention control measures.” |
| 3. Abstract: Transmission through inhalation is not reported as a single route. Why you suggested to wear medical mask to reduce transmission. | Thank you for this comment.  The current interim WHO Guidelines for mpox recommend health care workers use a respirator when in contact with mpox patients.  This review was conducted to inform the next WHO Guideline, and the decision of the guideline panel was to continue recommendation of the use of a medical mask,  Given the review findings, a medical mask may be more appropriate than a respirator for health care workers. |
| 4. Introduction: No references are included. Why? | Thank you, our apologies for this referencing error. The references have now been entered into the introduction. |
| 5. Methods: Please check the exclusion criteria. How many exclusion criteria did you consider? Any of the criteria? | Thank you, we have added the following sentence to the exclusion criteria for clarity:  “Studies were excluded if any of the following criteria were met:….” |
| 6. Use reference for WHO Clinical Management and Infection Prevention and Control guideline | Our understanding is that a reference is requested here for the WHO Guideline Development Group itself, as we are not referencing the guideline product here.  This reference has been added after checking the format with this WHO Group. |
| 7. Provide a definition for “suspected case of mpox infection”. | The review now states that:” The WHO definition of a suspected case of mpox infection was used (WHO 2022)” within the inclusion criteria section. |
| 8. Page 8: Data synthesis: “What is the proportion of new incident cases…...”   Is it new cases or 2ndary cases?  Why no cohort studies were considered for “What is the infectious period of mpox, disaggregated by route of transmission?” | Thank you for this question. The term ‘new incident cases’ had been used to include all reported cases, both primary and secondary. We have after discussion taken the decision to change this term to ‘incident cases’. It was not consistently possible to confirm which, if any, index case (case first recognized) in a paper was a true primary case. Our definition of an incident case is an individual changing from a state of non-disease to disease over a specific period of time. This has been added to the review for clarity.  Cohort studies were eligible for inclusion as stated in the inclusion criteria for the transmission of mpox section methods:  “Type of study: any scientific article of any design including clinical and environmental sampling studies.” |
| 9. Page 9: These following sentences are mentioned in method section.   “No randomized controlled trials or observational comparative studies were identified that directly informed any of the IPC review questions. No studies concerning mpox-like viruses met the inclusion criteria.” Avoid repeated information. | Thank you, these repeated sentences have been deleted. |
| 10. Page 10: the footnotes c and d are not properly identified | We have made the footnote explanations clearer and checked the tables that the footnotes are identified.  We have standardized the footnotes for all summary tables. |
| 11. Page 11: the footnotes c and d are not properly identified | We have made the footnote explanations clearer and checked the tables that the footnotes are identified.  We have standardized the footnotes for all summary tables. |
| 12. Table 3, 4 and 5: It is better to mention the study number. | Table 4 and 5 have been removed and summarized narratively as we needed to reduce the number of tables overall. References have been added to all other tables where possible. |
| 13. Page 17: Inferred odds ratio^c^: 1 : Is it suggested by a study or is it your interpretation? If it its resulted from other study, please add reference. | Thank you  The inferred odds ratio of 1 is our interpretation.  We have updated the footnote to explain as follows:  “Review findings indicated that mpox was transmitted in almost all occasions by direct physical contact; there were very few cases of fomite transmission. The review team inferred that if all lesions were covered, direct physical contact with others is avoided, and a medical mask worn, there would be no difference between the intervention and comparator groups.“ |
| 14. Page 17: Which term is more appropriate? Incident cases or 2ndary cases? Human-human transmission is not always required for Incident cases. | Incident cases has been used as a term in order to include both primary and secondary cases and permitted us to consider the study authors’ conclusions on the route of transmission for both groups.  We included all cases where acquisition was via human-to-human transmission. It was not always possible to determine whether a primary case was truly the index case. Primary cases may have acquired the infection from human-to-human contact outside of the group they introduce the disease into. Primary cases in certain settings were also reported to have been acquired due to live or dead animal contact. |
| 15. Discussion: First paragraph: This is useful if you add some logics that support direct physical sexual contact increase the risk of mpox transmission. | Thank you, the following has been added:  Secondary household attack rates are estimated to be 10% overall (reference). Household contact is the commonest reported route of acquisition of infection amongst children, but sexual contact is the commonest reported route of transmission amongst adults (reference).  Marshall et al (reference) investigated exposures amongst 313 healthcare workers in different settings, noting duration and type of contact as well as personal protective equipment used. No cases of mpox resulted from a range of contacts including direct skin-to-skin contact with lesions and exposure to aerosol generating proceedures with or without FFP3/N95 masks. Most of these contacts were brief. It is difficult to distinguish between the risks posed by close physical contact and sexual contact. However, more skin exposure, contact between mucous membranes or duration of contact may increase risk of transmission. In the 2022 global mpox outbreak, primary lesions commonly occurring at sites of sexual contact e.g. genital/anorectal or oral lesions and clustering of lesions at those sites support the conclusion that direct sexual contact is an important route of transmission (reference). |
| 16. Discussion: no discussion for suggested interventions to reduce mpox transmission. Please add a paragraph for that. | Thank you. The implications for practice paragraph in the discussion has been edited to include intervention suggestions and references. |

| Comments from Reviewer provided in PDF | Author team response |
| --- | --- |
| It’d be better to keep the number of decimal points consistent throughout. | Thank you, this has been made consistent to one decimal place. |
| Add “,” to separate “alone” & “the impact” | This has been done, thank you. |
| For Keywords, only the concept of mpox was listed. To improve the searchability of the manuscript after publication, it would be helpful to list other concepts (e.g., control measures, systematic review) that need to be highlighted. | Thank you, further keywords have been added: infection prevention, infection control, transmission, systematic review. |
| Introduction | |
| For Introduction, there is a lack of in-text citations supporting the narratives. Please add references to this section. | Thank you, these have now been added. |
| “ Incidence in endemic areas of central and West Africa had been increasing rapidly over the past decades, coinciding with cessation of smallpox vaccination and eradication programmes.”  It seems this sentence provides an explanation to the increase of incidence in the former sentence. Please consider rewording this word to strengthen the connection between between the two sentences. | These sentences have been amended as follows:  “Historically, mpox occurred primarily in west and central Africa, with infection commonly reported in persons who had contact with probable animal reservoirs;,and cases of secondary transmission reported in household contacts. The rapid increased incidence in endemic areas of central and West Africa over the past decades coincides with the cessation of smallpox vaccination and eradication programmes. The 2022 mpox outbreak was associated with sustained human-to-human transmission that had not been previously described.” |
| Consider using present tense here as this is a common fact. | Thank you, this has been changed to present tense. |
| (fomite transmission)  Seemingly a comma is missing here | A comma has been added. |
| Objective | |
| Despite the objective of assessing effectiveness, the data extracted from the reviewed literature seem not to be sufficient in fulfilling this objective. Evaluating effectiveness usually implies comparison of an effect between groups, but no/few data were available. The authors may consider adapting the objective to match the available data extracted from the review. | Thank you for raising this point; a similar point was raised by the second peer reviewer.  We have amended the objective as follows:  “To make inferences regarding the effectiveness of respiratory interventions and case isolation measures in reducing or preventing the transmission of mpox on the basis of synthesis of available literature.” |
| Methods | |
| Methods section is too long to follow. The authors may refer to published systematic reviews of PLOS GPH for truncation and put secondary contents in appendix. | Thank you, the methods section has been reduced and amended. |
| Ventilation: Seemingly, this should be treated as a footnote given it provides a definition. | Thank you, this has been changed to a footnote in all relevant places. |
| Please end sentences consistently all with/without period. | A period has been added where previously omitted for consistency. |
| Review question 3. Does isolating a person with mpox until all lesions are fully healed versus not isolating reduce mpox infections?  Reading this review question, it seems the subjects in Intervention and Comparator below are reversed. If not, please consider strengthening the wording of this question to strengthen its readability. | Thank you for this raising this important point.  The comparator, physical isolation of a person with mpox until lesions heal, was/is the current WHO guidance and is thus the comparator in this instance. It is compared to the potential new recommendation (intervention) of a patient with mpox not having to physically isolate provided all lesions are covered and a mask is worn ( a less restrictive option).  The intervention and comparator are in the correct position in this regard.  This section has been re-worded to increase readability. |
| Inclusion Criteria | |
| Population infected by mpox-like viruses are included in the review. It seems in results that the mpox-like population was conflated with the population with mpox infection. It’d be helpful to provide the rationale of why populations with mpox and mpox-like infections can be considered together for evidence synthesis. | Thank you for raising this point, we would like it to be understandable to all readers.  Cases of mpox-like infection were eligible for inclusion in the review. This participant group was prospectively decided to be eligible for inclusion in the predicted circumstance of scarce data for mpox-only infections.  After following the study selection process, there were no included studies including cases of mpox-like infection in the review.  There is a statement to this effect in the results section under “included studies” to make this clear to readers as follows:  “No studies concerning mpox-like viruses met the inclusion criteria.”  All tables have been checked to ensure the population states participants have confirmed mpox infection. |
| Selection of Studies | |
| The “Selection of Studies” section here seems to have the same content as the section with the same name next page despite paraphrasing. Please combine them to avert redundancy. | Thank you, this section has been amended. |
| Garritty 2020 - This citation, supposed to be for Rapid Review (RR) but not Systematic Review, was cited as a guidance for this Systematic Review. If this manuscript did follow a RR approach, the authors should consider replacing the mentioning of “systematic Review” throughout the manuscript with this approach. Else, justifications of why this approach for RR was used for this systematic review should be provided.  Compared to Garritty 2020 which is a presentation slides, the following peer-reviewed article seems to be a better reference here - https://www.jclinepi.com/article/S0895-4356(20)31146-X/pdf | We have undertaken a systematic review using rapid review methods for the study selection and data extraction stages only as outlined in the methods of the review.  Our understanding is that a rapid review is a method or form of synthesis that accelerates the process of conducting a systematic review; that is, we used rapid methods to conduct a systematic review. These methods were selected due to the time pressures of the review; at the time mpox was a Public Health Emergency of International Concern and findings were reported to the WHO guideline development group. We have added a sentence to explain the use of rapid methods to this effect in the review:  “Screening of results was undertaken according to the Cochrane Collaboration’s Rapid Review Methods due to the time-sensitivity of the review findings to inform guideline development (7). Title and abstract screening of all studies identified in the literature searches was undertaken independently by multiple authors; one author was required to assess a study as eligible for full text screening; two authors were required to assess a study as requiring exclusion. Full text screening against the inclusion criteria was undertaken independently by multiple authors. One author was required to assess a study as eligible for inclusion to data extraction; two reviewers were required to assess a study for exclusion. Authors resolved disagreement at any stage by discussion.”  Thank you for spotting this - we have changed to this reference. |
| This section, along with the highlighted “Risk of bias assessment” and “Data synthesis” sections, are available for the 2nd review but not available for the first review. For contents in this section that also apply to the former review, please move it to somewhere above.  Page 7 | Thank you; we have amended the methods section in response to this comment and a previous comment concerning the length of the methods section in general.  The methods section has now been reduced; full information on the review questions in PICO format has been moved to an appendix / supplementary material.  The description of the two-stage nature of the review process has been amended to reduce duplication of methods sections. |
| Risk of Bias Assessment | |
| Although the authors reported no actions were taken for assessing risk of bias, a convincing rationale for why this was not taken should be provided as this assessment is an indispensable part for Systematic Review. | Thank you for raising this important point; we apologise for omitting to fully explain our rationale here and have now added this into the text along with a restructure of the methods section.  Our rationale for the first review stage (aimed for RCTs and comparative studies): “It was planned for two authors to independently conduct risk of bias assessments using the Cochrane risk of bias 2 Tool for included randomized controlled trials and the ROBINS-I tool for included non-randomized comparative studies, however no comparative trials were identified for inclusion.”  We have reviewed the methods for the second stage of the review (including non-comparative observational studies).  We are not aware of any pre-existing risk of bias tool applicable to this situation and these study designs. The bias inherent in the study designs is considered in the GRADE process for observational studies. As an author team, we therefore devised a series of questions to appraise the quality of included studies regarding their representativeness and comphrehensivenes of participants.  This is now entered into the text as follows:  “Appraisal of study quality:  Risk of bias assessments are related to study design. We did not identify any applicable pre-existing tool to assess the risk of bias in included case reports and case series. We therefore constructed and piloted a series of questions to appraise the quality of included case reports and case series covering aspects of representativeness and comprehensiveness of included participants (Appendix 2). Quality appraisal assessments were then undertaken independently by the review authors for all included studies; differences were resolved by discussion. “  The appraisal questions and appraisal judgements have been placed in the Appendix.  Results are summarised in the Results section as follows:  “Quality assessment of included studies  The quality of assessment results are available in Appendix 4. Studies generally differed in whether they undertook and reported active case finding and in the reporting of sufficient information to hypothesize route(s) of transmission of mpox virus.” |
| Results | |
| A key issue for the Results section is there is a lack of appropriate citations in both writing and tables. Please add citations during revision. | Thank you, all citations have been added. |
| The number of tables seems too many in this manuscript. Please consider merging tables by research questions and moving some of the secondary tables to appendix. | Thank you, we have reduced the number of tables in the manuscript by narratively summarising their results instead and by moving others to the supplementary material. |
| Results: Review questions 1 and 2  This current content seems to be just a summarization of the titles in the following two tables. It is necessary to narrate (or at least highlight) findings in the tables. This comment applies to the rest of the Result sections where results in tables were not narrated. | Thank you, we have presented the main findings in the text for each relevant part of the results section. |
| It is not a common practice to name table like this, the authors should consider renaming “Summary of Findings Table” to “Table”. | Thank you, all tables have been renamed to begin “Table…” |
| SOFT 1  While the outcome of Review Question 1 is infection, the outcome here is “transmission”, so it seems these two terms were used interchangeably by the authors. However, transmission usually describes the process of pathogen acquisition, while infection describes the endpoint. As host immunity can clear pathogen to reduce infection, these two terms aren’t necessarily the same. Since the aim here is to use transmission data to infer infection, the authors should base the inference on necessary assumptions on immunity. | Thank you for this point.  We have amended the text to either use of the term “infection” or “ infection as a result of transmission” where appropriate. |
| SOFT 1 moderate rating  If the categorization here was based on the GRADE approach, how can the quality of evidence here be “moderate” without an observed effect being found in the reviewed literature? Further, all studies reviewed are observational, which are considered providing low quality of evidence (see section 5.1.1 in https://gdt.gradepro.org/app/handbook/handbook.html). The bottom line is it’s challenging to contemplate how “Moderate” was assigned here. How the guidance was used/adapted to grade the evidence should be articulated. | Thank you for this important point.  GRADE is not intended as a rigid system that does not permit making logical inferences in unusual situations.  For instance, one of the GRADE guidance papers published in the Journal of Clinical Epidemiology (included as a reference) <https://pubmed.ncbi.nlm.nih.gov/21802902/>  includes the following:  *Another situation allows an inference of a strong association without a formal comparative study. Consider the question of the impact of routine colonoscopy vs. no screening for colon cancer on the rate of perforation associated with colonoscopy.*  *Here, a large series of representative patients undergoing colonoscopy will provide high-quality evidence on the risk of perforation associated with colonoscopy. When control rates are near 0 (i.e., we are certain that the incidence of spontaneous colon perforation in patients not undergoing colonoscopy is very low), case series of representative patients (one might call these cohort studies of affected patients if they include large numbers of patients) can provide high-quality evidence of adverse effects associated with an intervention, thereby allowing us to infer a strong association from even a limited number of events.*  In this case, we found compelling evidence from observational studies that mpox was transmitted, in almost all occasions, by direct physical contact.  We further found compelling evidence that mpox transmission by inhalation did not occur, or if it did was extremely unusual.  Observational studies provided high certainty of this conclusion (analogous to the above example – in an observational study of a substantial number of representative patients, if a particular event never or almost never occurs, the strong inference that the event occurs rarely or not at all is warranted).  It is then a very compelling logical inference that if there are no or almost no cases of respiratory transmission, use of a respirator will not prevent any (or at the very least, very, very few) mpox transmissions.  That evidence is therefore at least moderate certainty of little or no benefit in preventing transmission from respirator use.  We have endeavoured to amend the Certainty of Evidence sections and summary of results tables to make our use of this GRADE process clear to the reader including adding the relevant reference to the paper in the JCE. |
| SOFT 1 footnotes p10  Seemingly, the odds ratio drawn from the inference is a little overstretching. An association that wasn’t observed does not mean this association doesn’t exist. The authors have stated that the effects for the intervention and comparator remain to be uncertain, so at least the high uncertainty of the inferred effect should be mentioned here.  Also, clarification should be made for whether the highlighted sentence was caused by no data were found through the literature search, or by the fact that some reviewed studies have concluded this. | Thank you, we have amended the review and endeavoured to transparently describe our methods and logic in the main text and footnotes regarding the odds ratio inference. As we have stated in response to the previous comment, we do not have high uncertainty in the inference that a respiratory intervention will have little or no impact on transmission when very little or no transmission occurs by the respiratory route. We have at least moderate certainty in the inference.  Thank you, the sentence has been clarified to reflect that this statement reflects the review findings in all relevant tables. |
| SOFT 2 same comments as for SOFT 1 as per three boxes above | Thank you, our response is the same as outlined in the SOFT 1 comment box; the logic of the inference is not different for this comparison. |
| Table 4: incident cases by route of transmission | |
| It’d be helpful to articulate on footnote on how the denominator was calculated. | Thank you, all footnotes have been amended to state how the denominator was calculated |
| Please provide citations to these studies. | Citations have been added |
| Table 5 | |
| If this is the timing when the naming of the new clade became available, seemingly it’d be better to add to the table’s footnote that 2022 in the table refers to August 2022. | Thank you, we have amended the footnotes to better explain the categories. |
| It may be better to articulate on footnote on how the denominator was calculated for this and other columns on this table. | These footnotes have been added |
| Table 6 | |
| The margin of a column usually adds to 100% across categories, so the rest of the 2.5% need to be clarified. Is this remaining 2.5% the same as the 2.5% in the right column? If so, it’s a bit challenging to interpret as the 97.5% includes all the three categories, while the 2.5% in the right column includes the last two categories - couldn’t these two columns be merge into one column?  Please better align the above horizontal line to have this proportion solely corresponds to fomite & inhalation. | Thank you, this table has now been deleted and the results summarised in the text.  (We were asked to reduce our overall number of tables.) |
| Table of mpox in household setting | |
| Since the numbers across categories in the table cannot be distinguished, I suggest removing this table. | Thank you, we have deleted this table; the narrative summary of results relating the table remains. |
| Table: Clinical samples of viral isolation attempts from adults or children with confirmed mpox  infection | |
| As mentioned, please provide references to the tables | References have been added, thank you |
| The two studies (Hernaez 2022, Mileto 2022) for this table were both conducted in Europe (Italy/Spain). How these countries were shown here. | Thank you for spotting this error; this has been amended. |
| Clinical and environmental sampling: review question 1 and 2 | |
| Authors identified two studies in which viral isolation was attempted from air samples collected in  rooms occupied by an individual with mpox (Hernaez 2022, Gould 2022).  Does this sentence mean those rooms were only occupied by an infected individual in each room, or were occupied by an infected individual with others? Please clarify. | Thank you  This sentence has been edited for clarity. |
| Which study? Please clarify by adding a citation. | This citation has been added |
| Table: : Air sampling in environments occupied by adults with confirmed mpox infection | |
| As percentages were reported in other tables. Seemingly, it’d be good to report them here to maintain consistency among tables. | Thank you, percentages have been added |
| Review question 1 and 2: subgroup, patient to patient transmission | |
| “there was no known physical contact  between the two. Authors hypothesized fomite or inhalation transmission.”  Please consider better indicating who (the authors of that reviewed article or the authors of this manuscript) was making the hypothesis. | Thank you, this has been amended to state “review authors…” to reflect we made the hypothesis. |
| “there was no known physical contact  between the two. Authors hypothesized fomite or inhalation transmission.”  Others could argue that correlation does not imply causation. E.g., these children could be separately infected elsewhere and be at the hospital later. So, please provide more context here to help readers understand the rationale of this hypothesis. | Thank you, we have provided more context of the case here to aid clarity and to make our transmission hypothesis more transparent. |
| Certainty of the evidence  As mentioned previously, the exact meaning of “moderate” here remains hanging in the air without explanation of how the GRADE approach was implemented in this manuscript. | Thank you, we have addressed similar comments above and amended the review text accordingly to explain this clearly to readers as it is an unusual situation. |
| SOFT 3 | |
| Comparing this table with Summary of Findings Tables 1 & 2, how can the characterizations of the certainty of evidence, based on uncertain effects estimates, differ between this and the former two tables? I suggest the authors provide their understandings/definitions of these certainty levels. | The first two SOF tables dealt with respiratory protection measures. The third SOF deals with a different intervention, physical isolation. It is natural that the certainty of the evidence will differ for different interventions and comparators.  The first two SOFs concern measures to protect against respiratory transmission. Since the evidence demonstrates that respiratory transmission almost never occurs, we have at least moderate certainty evidence that respiratory protection measures will have little or no effect.  The third SOF deals with physical isolation. Since transmission occurs by direct contact, covering of lesions until healed should prevent infection. We found that logical inference less compelling than the inference regarding the lack of benefit from respiratory measures when respiratory transmission rarely or never occurs. That is the reason for the difference in certainty of evidence. |
| Review question 3 | |
| Incident cases by route of transmission  As mentioned, please provide references for data summarized in Tables 2 and 3. | References have been added, thank you |
| Clinical and environmental samples | |
| Please also have these references reflected in the below table | This has been added. |
| Table 8: Mpox lesion clinical samples in which viral isolation was attempted | |
| For consistency, it’d be helpful to keep the number of digits the same within and between tables. | The number of digits has been made consistent in all tables |
| Clinical and environmental samples | |
| Seemingly, it would be of readers’ interest to further delineate the types of environmental surface that were sampled, as this could inform future interventions. | Thank you, further details have been added.  The paragraph now reads:  “Five studies attempted viral isolation from environmental surface samples (73, 128, 132-134). Each study sampled high-touch surfaces (for example door handles and switches) and items that had been in close contact with infected persons (including towels and clothes). Studies conducted within hospitals included sampling of the anterooms in which personal protective equipment was doffed and disposed, and sampling of the personal protective equipment (128,133). Gould et al 2022 (128) included sampling of a deposition area in each room which was unlikely to have been touched by patients or staff. Morgan et al 2022 (73) compared the frequency and quantity of virus detection from samples of non-porous and porous articles; authors reported that detection of viable virus was significantly more frequent from samples collected from porous materials. The frequency of detection of replication competent virus was between 0 and 60% in surface samples (Table S8). “ |
| Discussion | |
| “There is no evidence….”  It seems this statement is too absolute. Changing the writing to “There is no observed evidence” or “We did not observe evidence…” may be better. | We have changed this to:  “We found no evidence…” |
| Strengths and limitations  In this work, non-English articles are excluded from the review, which may bias the findings. So this could be mentioned here.  Many limitations explained here could be caused by publication bias, so it would be great to mention this here. | Thank you for this comment.  This has now been mentioned as a limitation.  Thank you, we acknowledge that publication bias may influence the completeness of the collected evidence.  Our understanding is that the publication system is sensitive to the review topic of transmission routes (that is, if a route of transmission is found, it is likely to be published. If it is not found, it is unlikely to be published). In this sense we would expect bias towards publication of route of transmission data and would not be a substantive bias for us missing significant transmission route frequency.  We have amended the discussion section with these points. |
| Implications for practice and research | |
| “Epidemiological evidence and data from clinical and environmental sampling does not support the hypothesis that respiratory or fomite modes of transmission are significant.”  A lack of evidence found does not necessarily mean this, and this could also be caused by publication bias. The authors could address this by making the tone of this sentence less assertive and definitive. | Thank you, this sentence has been amended to:  “Epidemiological evidence and data from clinical and environmental sampling provides limited support for the hypothesis that respiratory or fomite modes of transmission are significant.” |
| Other References | |
| It’s uncommon to have a separate reference section, please consider merging this section with the previous reference section. | Thank you the sections have been merged. |
| Search strategy | |
| Although mentioned in the main text, it would be helpful to specify here the publication year range of the articles reviewed. | This has been added into this section where it was missing for Web of Science and GIM databases |
| Please clarify the association among these bullets of search strategy. | Line 4 combines the three previous searches , where we searched for the concept of “monkeypox” using controlled vocabulary (Monkeypox virus/ or Monkeypox/, line 1 ), or as a text word in the title and abstract fields, using two different versions of the name – lines 2 and line 3). |
| Please clarify the association among these bullets (bullets 1-19) of search strategy. | This search is for Mpox-like viruses. Line 3 combines the searches for Mpox-like viruses using controlled vocabulary (line 1) or text terms (line 2). Lines 8-11 (joined in line 12) report terms for ventilation and air quality , and line 14 combines the search results for Mpox-like viruses AND line 12. Similarly, line 18 combines results for Mpox like viruses AND hospital transmission. Line 19 joins all results from lines 12 + line 18 ie the concepts of Mpox-like viruses and ventilation or air quality or hospital transmission. |
| Could you clarify which section above these search strategies correspond to? | Line 4 combines the results for Mpox-like viruses (line 1) plus the results for terms for ventilation and air quality (line 2). Line 5 combines the search results for Mpox-like viruses (line 1) AND hospital transmission. (line 3). Line 6 joins all results from lines 4 + line 5. |
