# Supplementary material for: Infection prevention and control measures to reduce the transmission of mpox: A systematic review
Source: PLOS Glob Public Health. 2024 Jan 18;4(1):e0002731. doi: 10.1371/journal.pgph.0002731 (PMC10796032; doi:10.1371/journal.pgph.0002731)
Supplement: Attachment — Submitted filename: Response to Reviewers.docx [file pgph.0002731.s018.docx]

**Title:** Infection prevention and control measures to reduce the transmission of mpox: a systematic review

**Response to Reviewers**

| **Reviewer’s comment** | **Authors’ Response** |
| --- | --- |
| **Abstract, referring to (0.0%)**  This is unclear and needs to be revised. | Thank you for your comment. To clarify, the 0.0% value is referring to the fraction preceding it, 2/4309, expressed to one decimal place. Previous peer review comments requested that all percentages be standardised to the same number of decimal places.  In response to this comment, the author team is okay with this percentage being removed completely, or written to zero or two decimal places, as per PLOS style preference. |
| **Introduction**  The following sentence is in the response letter but not the revised manuscript - “The 2022 mpox outbreak was associated with sustained human-to-human transmission that had not been previously described.”. Please ensure the contents are aligned in different documents submitted. | Thank you, and we have checked the manuscript version history.  To confirm, the sentence “The 2022 mpox outbreak was associated with sustained human-to-human transmission that had not been previously described” is the third sentence in the introduction of the revised manuscript. |
| **Introduction**  Seeks?  In reference to “seeking” | We have amended this to “seeks”, as suggested by the reviewer. |
| **Review Questions**  “Does healthcare worker use of respirator versus a medical mask when interacting with a  confirmed/suspect mpox patient during the infectious period reduce mpox infections?” | We have selected the term “worker” rather than “worker’s” to reflect the intention of general healthcare worker use rather than an individual worker’s use. |
| **Review Stage One**  Is words like “related to” missed here?  In reference to: “In the first stage, we aimed to synthesize evidence concerning the review question interventions from available interventional comparative studies.” | Thank you for your comment. We have selected the term “concerning” rather than “related to” in this sentence. We feel it conveys the same meaning, and saves one word. |
| **Methods, inclusion criteria, population**  What is this? Has this abbreviation been defined yet?  In reference to “MPX” | Thank you for spotting this. We have changed this term to “mpox” in the population section of the inclusion criteria for review stage one and review stage two.  We performed the Find function to search for any other overlooked text including “MPX” – no further occasions were found. The correct term now is mpox. |
| **Results, included studies**  I understand the authors’ effort in making the reference list, but a long reference list like this is just too hard to follow. If references are mentioned in a table, the author can replace the references with a citation to the table, which could save lots of space. | Thank you for this comment. We have endeavoured to be thorough with the referencing and appreciate the cumbersome nature of this referenced section.  The reference list commented upon here, concerning studies conducted prior to the mpox 2022 outbreak and studies published during the 2022 outbreak, are not referenced in this division in any table, and so a citation to a Table as suggested is not possible. Similarly, the references for country of acquisition of infection are not referenced within a table in this division.  All included studies are referenced in the first sentence of the “included studies” section. Our preference is to keep the references listed within this section, but are willing to amend in line with PLOS style, if needed. For readability, we have shortened the references for consecutive references, e,g “105-110” instead of “105, 106, 107, 108, 109, 110” in the manuscript.  . |
| **Results, review question 1 and 2**  As suggested in the first round, if tables are reported, some illustration for them are usually expected. I cannot find writings for Tables 2 and 3 in this section. | Thank you, and we have now entered text referring to tables 2 and 3 as follows:  “The summary of findings for review question 1 are presented in Table 2 and the summary of findings for review question 2 are presented in Table 3. “ |
| **Table 3: Summary of findings: Airborne precaution room versus an adequately ventilated room in a**  **healthcare facility for a mpox patient for reducing mpox infection**  Just a single patient? | Thank you, we have changed this to:  “Table 3: Summary of findings: Healthcare facility use of an airborne precaution room versus an adequately ventilated room for mpox patients for reducing mpox infection.” |
| **Number of incident cases by route of transmission**  Since Table 4 is cited and the same references are in the table, the reference here can be removed. | Thank you, we have removed these references accordingly. |
| **Certainty of the evidence**  I may have missed this - can the authors explain how “logical inferences” were conducted?  Referring to: “The certainty of the evidence for review questions 1 and 2 are based on logical inferences in this situation in which a formal comparison is lacking.” | Thank you for this comment, we want this point to be clear to readers.  The explanation underlying the logical inference is explained following the sentence highlighted in the rest of the paragraph.  To aid clarity, we have moved the opening sentence to the end of the paragraph. In this way, the explanation starts and builds through the paragraph, ending now with the statement that the certainty of evidence has been made using logical inferences. References to the GRADE guidance in this situation are made within the paragraph. The new paragraph reads as follows for review questions 1 and 2:  “Certainty of evidence commenced at a rating of low due to inclusion of observational study designs (12). Evidence from observational studies indicated that mpox was transmitted, in almost all occasions, by direct physical contact.  We further found compelling evidence that mpox transmission by inhalation did not occur, or if it did, was extremely unusual.  For review question 1, the logical inference was made that if there are no or almost no cases of transmission by inhalation, use of a respirator by a health care worker would prevent either none or very few mpox transmissions (Table 2). The evidence is therefore at least moderate certainty of little or no benefit in preventing transmission from respirator use. Similarly, for review question 2, the evidence is at least moderate that the use of an airborne precaution room probably has little to no impact on preventing mpox transmission (Table 3). The certainty of the evidence for review questions 1 and 2 are thus based on logical inferences in this situation in which a formal comparison is lacking (12, 135).”  And for review question 3:  “Certainty of evidence commenced at a rating of low due to inclusion of observational study designs (12). Evidence from observational studies indicated that mpox was transmitted, in almost all occasions, by direct physical contact. There were very few cases of fomite transmission. The logical inference was made that there may be minimal added benefits to physically isolating cases provided all lesions are covered, direct physical contact with others is avoided and a medical mask is worn (low to moderate certainty, downgraded one to two levels for serious risk of indirectness; Table 6). The certainty of the evidence for review question 3 is based on a logical inference in this situation in which a formal comparison is lacking (12,135).” |
| **Discussion**  This needs to be revised  (In reference to the 0.0%) | Thank you.  As noted above, similarly to the abstract, the 0.0% is referring to the fraction preceding it, 2/4309, expressed to one decimal place. Previous peer review comments requested all percentages be standardised to the same number of decimal places.  We have removed this 0.0% value from this sentence completely, or can amend to zero or two decimal places, as per PLOS editorial preference. |
| **Discussion, strengths and limitations**  A scoping review is usually conducted prior to a full-scale systematic review (SR) to ensure there is sufficient literature for the SR. I wonder why the authors conducted the scoping reivew after the SR is done. Please clarify. | Thank you, our use of the term scoping search here is misleading and may confuse readers.  We have altered the sentence to: “We are not aware of any systematic reviews addressing the same review questions covering the same scope or with as comprehensive inclusion criteria since the date of our literature search.” |
| **Other** | We have changed the term “respiratory” to “airborne and droplet” where applicable to reflect the World Health Organization nomenclature for infection prevention and control measures.  The word “home” has also been added where applicable when referring to the intervention of physical case isolation for reader clarity. |
| **Other** | Other small terminology edits have been made with tracked changes. |
